# Abscisic acid regulates Cl^−^ efflux via the ABI5-ZAT10-SLAH3 module in chloride-stressed *Malus hupehensis*

**DOI:** 10.1093/hr/uhae200

**Published:** 2024-07-24

**Authors:** Jianfei Song, Junhong Yan, Baozhen Sun, Bing Chen, Xiaoyue Zhu, Hongcai Wei, Zhilong Bao, Fangfang Ma, Weiwei Zhang, Hongqiang Yang

**Affiliations:** College of Horticulture Science and Engineering, Shandong Agricultural University, Tai’an, 271018, Shandong, China; Apple Technology Innovation Center of Shandong Province, Tai’an, 271018, Shandong, China; College of Horticulture Science and Engineering, Shandong Agricultural University, Tai’an, 271018, Shandong, China; Apple Technology Innovation Center of Shandong Province, Tai’an, 271018, Shandong, China; College of Horticulture Science and Engineering, Shandong Agricultural University, Tai’an, 271018, Shandong, China; Apple Technology Innovation Center of Shandong Province, Tai’an, 271018, Shandong, China; College of Horticulture Science and Engineering, Shandong Agricultural University, Tai’an, 271018, Shandong, China; Apple Technology Innovation Center of Shandong Province, Tai’an, 271018, Shandong, China; College of Horticulture Science and Engineering, Shandong Agricultural University, Tai’an, 271018, Shandong, China; Apple Technology Innovation Center of Shandong Province, Tai’an, 271018, Shandong, China; College of Horticulture Science and Engineering, Shandong Agricultural University, Tai’an, 271018, Shandong, China; Apple Technology Innovation Center of Shandong Province, Tai’an, 271018, Shandong, China; College of Horticulture Science and Engineering, Shandong Agricultural University, Tai’an, 271018, Shandong, China; College of Horticulture Science and Engineering, Shandong Agricultural University, Tai’an, 271018, Shandong, China; College of Horticulture Science and Engineering, Shandong Agricultural University, Tai’an, 271018, Shandong, China; Apple Technology Innovation Center of Shandong Province, Tai’an, 271018, Shandong, China; College of Horticulture Science and Engineering, Shandong Agricultural University, Tai’an, 271018, Shandong, China; Apple Technology Innovation Center of Shandong Province, Tai’an, 271018, Shandong, China

## Abstract

The overload of Cl^−^ typically causes cell damage and death in plants, especially in Cl^−^-sensitive crops. Abscisic acid (ABA) is a stress-induced phytohormone that can alleviate chloride stress by reducing Cl^−^ accumulation; however, the mechanism is not clear. Here, we found that the application of ABA elevated Cl^−^ efflux from roots and reduced membrane damage and cell death in chloride-stressed *Malus hupehensis*. *MhSLAH3*, a homolog of the slow anion channel from *M. hupehensis*, encoded a channel controlling Cl^−^ efflux and was induced by both chloride and ABA. *MhSLAH3* overexpression accelerated Cl^−^ efflux, which enhanced the tolerance of *M. hupehensis* to chloride stress, and retarded chloride-induced cell death. However, the suppression of *MhSLAH3* partially offset the acceleration effect of ABA on Cl^−^ efflux. MhZAT10L was then identified as a C2H2-type transcription factor upstream of *MhSLAH3*, repressing *MhSLAH3* transcription under chloride stress. The suppression of *MhZAT10L* accelerated Cl^−^ efflux by releasing suppressed *MhSLAH3*, but *MhZAT10L* overexpression counteracted the effects of ABA on Cl^−^ efflux. MhABI5 promoted Cl^−^ efflux mediated by *MhSLAH3* due to induction by ABA and transcriptional repression of *MhZAT10L*, but this function of MhABI5 was reversed by *MhZAT10L* overexpression. The suppression of *MhABI5* diminished the positive effects of ABA on Cl^−^ efflux and retarding cell death. Thus, ABA repressed *MhZAT10L* transcription by activating MhABI5, further releasing *MhSLAH3* to accelerate Cl^−^ efflux. These findings provide a new evidence of ABA regulation of Cl^−^ efflux.

## Introduction

Chlorine is a beneficial and essential micronutrient for plants [1], and only 50–100 μM chlorine is needed for healthy plant growth [2]. In soil, it primarily takes the form of chloride (Cl^−^ salinity, abbreviated as Cl below), such as NaCl, KCl, CaCl_2_, and MgCl_2_. Cl in soil is often excessive due to the applications of either animal wastes rich in anions of chlorine (Cl^−^) or atmospheric depositions [3]. However, in prior reports on salinity stress, most scholars have focused on osmotic stress or cation toxicity, such as Na^+^, with little attention given to Cl^−^ toxicity [4, 5].

The optimal Cl^−^ demand for satisfactory plant growth falls within the 25.0–50.8 mg·g^−1^ dry weight (DW), but for Cl^−^-sensitive plants (i.e., grapevine, citrus, apple, soybean, pear, and strawberry) only requires Cl^−^ within the 0.3–10.0 mg·g^−1^ DW [6]. Once beyond this range, excessive Cl^−^ has toxic effects, such as destroying photosynthetic electron receptors and transport chains, inducing the overaccumulation of reactive oxygen species (ROS), damaging the cell membrane, and even causing cell death [3, 6]. The overload of Cl^−^ in cells also causes cell senescence by inhibiting nutrition anion absorption, namely, NO_3_^−^ and H_2_PO_4_^−^, and cytoplasmic enzyme activities such as ribosomal enzyme and nitrate reductase activity [6–9]. The excessive supply of Cl^−^ inhibits polyphenol oxidase activity and reduces coffee quality [10]. The negative effects of excess Cl^−^ are more pronounced in Cl^−^-sensitive plants. The excess Cl^−^ in citrus leaves greatly reduces photosynthesis and stomatal conductance, leading to a decrease in DW and an increase in defoliation [11]. *Malus hupehensis* is often used as apple rootstock, but it is susceptible to Cl^−^. The overaccumulation of Cl^−^ in its roots leads to severe membrane damage and increased ROS generation [12], but it can be alleviated by increasing endogenous abscisic acid (ABA) [13]. Nevertheless, the mechanism through which ABA controls Cl^−^ accumulation is presently not understood.

Cl^−^ enters roots through both symplastic and apoplastic pathways, leading to Cl^−^ accumulation. In high-Cl^−^ conditions, this process is generally passive due to a membrane potential that is more positive than the equilibrium potential for Cl^−^ [14]. However, Cl^−^ efflux is proactive and primarily mediated by slow anion channels (SLAC), Cl^−^ channels (CLC), and aluminum-activated malate transporters (ALMT transporters) [14, 15]. *SLAC1* overexpression in tobacco BY-2 cells elevates cryptogein-induced Cl^−^ efflux [16], but *MhCLC-c1* suppression enhances NaCl sensitivity and intracellular Cl^−^ accumulation in apple calli [17]. Among the Cl^−^ efflux controllers, SLAC and its associated homologs (SLAH) play more important roles [15]. SLAC1, SLAH1, and SLAH3 participate in regulating the accumulation of Cl^−^, and SLAC1 regulates Cl^−^ efflux in the guard cells of Arabidopsis [18], while SLAH1 modulates Arabidopsis Cl^−^ accumulation and long-distance Cl^−^ transport [19, 20]. SLAH1 also gates SLAH3, which is open for Cl^−^ translocation from root to shoot [21]. PttSLAH3 in poplar functions independently of protein kinase-mediated phosphorylation to absorb Cl^−^ [22], and the shoot Cl^−^ efflux and salt tolerance of grapevine are linked to *SLAH3* expression [23]. The expression of *AtSLAH3* is controlled by endogenous ABA and further decreases Cl^−^ accumulation [13].

A prior report has demonstrated that exogenous application of ABA inhibits Cl^−^ absorption [24]. Increasing endogenous ABA levels can also inhibit Cl^−^ accumulation [13], but downregulating endogenous ABA reduces Cl^−^ exclusion from the root apex and decreases salt tolerance [25]. ABA signaling regulates Cl^−^ accumulation in the roots of Cl-tolerant grapevine rootstocks via SNF1-related protein kinases (*VvSnRK2*.*6* and *VvSnRK2*.*7*) [23]. Upon encountering stress signals, ABA-activated SnRK2 kinases phosphorylate ABA-INSENSITIVE 5 (ABI5) and subsequently regulate stress-adapted genes expression [26, 27]. ABI5 assumes a pivotal role in facilitating ABA-mediated stress responses [26]. ABI5 promotes heat-stressed chlorophyll degradation and upregulates fatty acid desaturation and flavonoid synthesis-related gene expression during cold stress after it is activated by ABA [28]. ABI5 also enhances aluminum tolerance by modulating cell wall modification and osmoregulation-related gene expression and alleviates iron deficiency by accelerating iron transport via ABA signaling [29, 30]. Hence, ABI5 may also be involved in the ABA-mediated Cl response.

The transcription factor ZAT10, a C2H2 zinc finger protein (ZFP) member, plays a pivotal role in the regulation of abiotic stress tolerance [31]. ZAT10 is the first ZFP related to salt stress, and it maintains ionic balance by modulating ion balance-related genes expression [31]. ZAT10 improves the freezing tolerance by affecting *PeAPX2* transcription in poplar [32] and CBF-dependent pathway in upland cotton [33]. MhZAT10 interacts with MhDREB2A, positively regulating cold and drought resistance [34]. In addition, ZAT10 negatively regulates abiotic tolerance. Overexpression of MdZAT10a-like decreases salt tolerance in apple by directly repressing *MdNHX1* expression, and this effect can be strengthened through interacting with MdbHLH100 [35]. MdZAT10, inhibited by ABA, increases drought sensitivity and accelerates leaf senescence by interacting with MdABI5 [36, 37]. PpZAT10 enhances peach cold sensitivity by repressing vacuolar invertase activity [38]. Our prior report also demonstrated that MhZAT10L from *M. hupehensis* promotes cell death under NaCl stress by activating *VPE* expression [39], but how it responds to Cl is unclear.

*M. hupehensis* Rhed. var. *pingyiensis* Jiang is widely employed as a rootstock for apple trees and Chinese flowering crabapple, providing roots for cultivating apples in actual production. We have previously found that it is susceptible to Cl^−^ [12], thus implying that it is a Cl^−^-sensitive rootstock, similar to grapevine and citrus. Here, we isolated MhZAT10L from *M. hupehensis*, a C2H2 TF that represses *MhSLAH3* transcription. We analysed its function in Cl^−^ efflux and further investigated the mechanism of ABA regulation of Cl^−^ accumulation and cell death through the ABI5-ZAT10 module in Cl-stressed *M. hupehensis*. These findings offer a fresh perspective on the mechanism by which ABA modulates the efflux of anions, especially Cl^−^.

## Results

### Abscisic acid regulates Cl^−^ efflux and alleviates chloride-induced cell death

**Figure 1:**
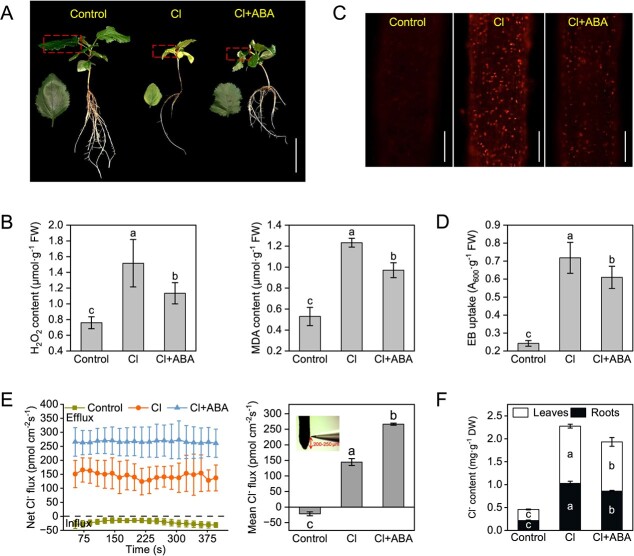
Effect of abscisic acid on cell death and net Cl^−^ flux in roots of chloride-stressed *Malus hupehensis*. **A** Phenotype of *M. hupehensis* under 50 mM chloride (Cl^−^-containing salts, abbreviated as Cl below) and 50 mM Cl treatment with the addition of 10 μM abscisic acid (ABA). Bars, 5 cm. **B** H_2_O_2_ and MDA content of *M. hupehensis* roots under 50 mM Cl and 50 mM Cl with the addition of 10 μM ABA. **C** Assessment of propidium iodide (PI) fluorescence intensity for identifying the dead cells distribution in *M. hupehensis* roots under 50 mM Cl and 50 mM Cl with the addition of 10 μM ABA. Bars, 100 μm. **D** Determination of Evans blue (EB) uptake value to measure the rate of cell death in *M. hupehensis* roots under 50 mM Cl and 50 mM Cl supplemented with 10 μM ABA. **E** Net Cl^−^ flux. **F** Cl^−^ content of *M. hupehensis* under 50 mM Cl and 50 mM Cl with the addition of 10 μM ABA. The data are shown as the mean ± standard deviation (*n* = 3). Statistically significant differences (*P* < 0.05) are indicated by different letters in each column (one-way ANOVA).

Our prior data indicated that increasing endogenous ABA reduced Cl^**−**^ accumulation [13]. Here, in contrast to the damage caused by high concentrations of ABA (≥20 μM ABA for *M. hupehensis* and apple calli; ≥2 μM ABA for Arabidopsis), applying 5–10 μM of exogenous ABA to *M. hupehensis* and apple calli, as well as applying 0.1–1 μM ABA to Arabidopsis, under Cl stress improved growth inhibition (Fig. 1A; Fig. S1, see online supplementary material). The ABA also reduced the hydrogen peroxide (H_2_O_2_) and malondialdehyde (MDA) content in roots of Cl-stressed *M. hupehensis* (Fig. 1B). Compared to the control group, Cl stress resulted in increased roots cell death, as indicated by propidium iodide (PI) and Evans blue (EB); however, both the fluorescence intensity of PI and EB uptake significantly decreased after the addition of exogenous ABA (Fig. 1C). Non-invasive Micro-test Technology (NMT) evidenced that the roots displayed Cl^−^ efflux under Cl stress, and the Cl^−^ efflux rate further increased after ABA application (Fig. 1D). The Cl^−^ content of roots and leaves decreased after ABA addition (Fig. 1E). Overall, ABA promoted Cl^−^ efflux from roots and alleviated Cl^**−**^ accumulation and Cl-induced cell death.

### Abscisic acid regulates Cl^−^ efflux and chloride-induced cell death through MhSLAH3

**Figure 2:**
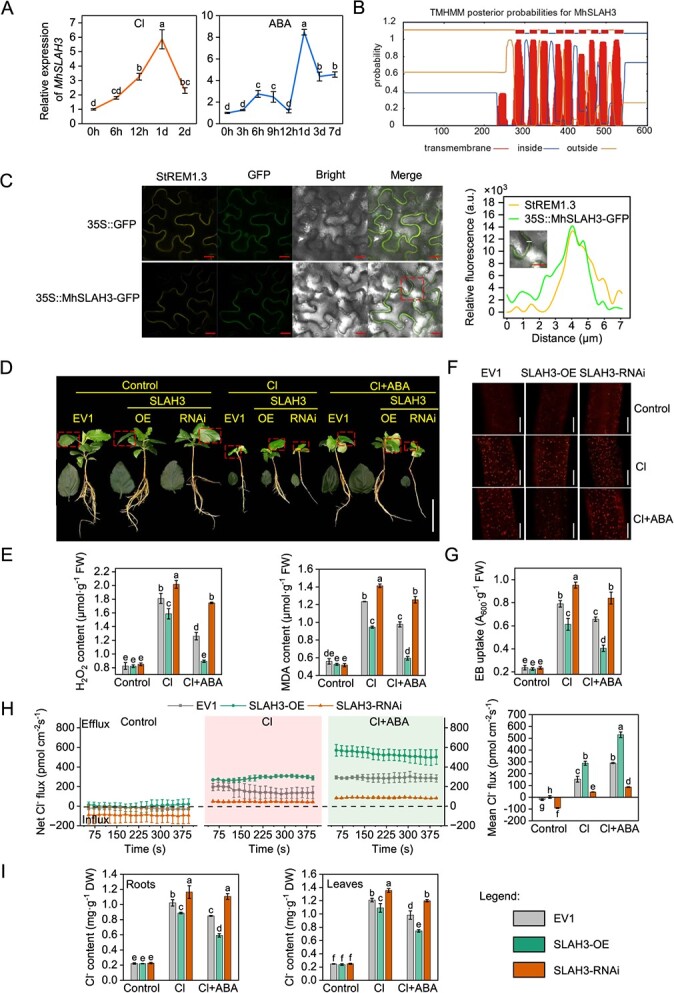
Effects of abscisic acid on cell death and net Cl^−^ flux in *MhSLAH3* transgenic hairy roots of *Malus hupehensis* under chloride stress. **A** Relative expression of *MhSLAH3* under treatment with 50 mM chloride (Cl) and 10 μM abscisic acid (ABA). **B** Transmembrane domain prediction. **C** Subcellular localization. StREM1.3, a protein located in the plasma membrane, was used to label the plasma membrane. The comparable fluorescence intensity of 35S::MhSLAH3-GFP and StREM1.3 at identical sites suggests their shared subcellular localization. Bars, 20 μm. **D** Phenotypic comparison between *MhSLAH3*-overexpressing (SLAH3-OE), empty expression vector (EV1), and *MhSLAH3*-suppressing (SLAH3-RNAi) hairy root *M. hupehensis* seedlings under 50 mM Cl and 50 mM Cl with 10 μM ABA application. Bars, 5 cm. The phenotypic data are from one representative line out of at least three transgenic lines of SLAH3-OE or SLAH3-RNAi. **E** H_2_O_2_ and MDA content. **F** Distribution of dead cells of SLAH3-OE, EV1, and SLAH3-RNAi roots under 50 mM Cl, and 50 mM Cl with 10 μM ABA application. Bars, 100 μm. **G** Cell death rate. **H** Net Cl^−^ flux. **I** Cl^−^ content of SLAH3-OE, EV1, and SLAH3-RNAi under 50 mM Cl and 50 mM Cl with 10 μM ABA application. The data are shown as the mean ± standard deviation (*n* = 3). Statistically significant differences (*P* < 0.05) are indicated by different letters in each column (one-way ANOVA).

The slow anion channels (SLAC/SLAH) are known for their high permeability to Cl^−^ and their significant involvement in Cl^−^ accumulation, long-distance transport, and translocation [15]. We identified four SLAC/SLAH family members (one MdSLAC and three MdSLAHs) in the apple proteome, conducted phylogenetic analysis (Fig. S2A, see online supplementary material), and analysed the tissue expression patterns of their homologs in *M. hupehensis*. Unlike the high expression patterns of *MhSLAC1*, *MhSLAH1*, and *MhSLAH2* in the leaves, *MhSLAH3* was strongly expressed in the roots (Fig. S2B, see online supplementary material) and significantly induced by Cl and ABA (Fig. 2A); therefore, *MhSLAH3* was selected for further investigation.

MhSLAH3 from *M. hupehensis* exhibited a high level of similarity (98.83%) to MdSLAH3 (Table S3 and Fig. S3, see online supplementary material). The transmembrane hidden Markov model (TMHMM) predicted that it contained 10 transmembrane domains (Fig. 2B). Subcellular localization assays revealed that MhSLAH3 was located in the plasma membrane (PM) (Fig. 2C).

The *35S::MhSLAH3-GFP* (SLAH3-OE) and *35S::antiMhSLAH3-GFP* (SLAH3-RNAi) fusion vectors were introduced into the shoot base cells of *M. hupehensis* seedlings utilizing *Agrobacterium rhizogenes*-mediated infection to produce transgenic roots (Fig. S4, see online supplementary material). The hairy roots with an empty vector (EV1) transformation were employed as a negative control. Multiple SLAH3-RNAi, EV1, and SLAH3-OE lines were thus obtained (Fig. S5, see online supplementary material), and they showed similar growth under normal conditions (Fig. 2D); however, the SLAH3-OE lines had better growth and lower root H_2_O_2_ and MDA content than the EV1 and SLAH3-RNAi lines under Cl stress (Fig. 2E). In contrast, the SLAH3-RNAi lines exhibited poor growth and had the highest H_2_O_2_ and MDA content (Fig. 2D and E). The increased fluorescence intensity of PI and higher EB uptake values indicated that SLAH3-RNAi roots had the most dead cells (Fig. 2F). Unlike the Cl^−^ influx observed in the SLAH3-RNAi and EV1 lines, the SLAH3-OE lines showed slight Cl^−^ efflux under normal conditions (Fig. 2G). However, all of the seedlings exhibited Cl^−^ efflux under Cl stress (Fig. 2G). Among these, the SLAH3-OE lines showed the highest rate of Cl^−^ efflux and the lowest Cl^−^ content in the roots and leaves (Fig. 2G and H). In contrast, the SLAH3-RNAi lines had the lowest rate of Cl^−^ efflux and the highest Cl^−^ content (Fig. 2G and H).

The addition of exogenous ABA not only enhanced seedling growth, but it also resulted in a decrease in the H_2_O_2_ and MDA content, as well as a reduction in cell death (Fig. 2D–F). In addition, it accelerated Cl^−^ efflux and decreased the Cl^−^ content (Fig. 2G and H). However, the positive effects of ABA on SLAH3-RNAi lines, such as a significant reduction in cell death and a greater enhancement in the Cl^−^ efflux rate, were less prominent compared to SLAH3-OE and EV1 lines (Fig. 2D–H), suggesting that the absence of MhSLAH3 weakened the effect of ABA*.*

### MhZAT10L acts as a mediator in abscisic acid regulation of Cl^−^ efflux by modulating *MhSLAH3* transcription

**Figure 3:**
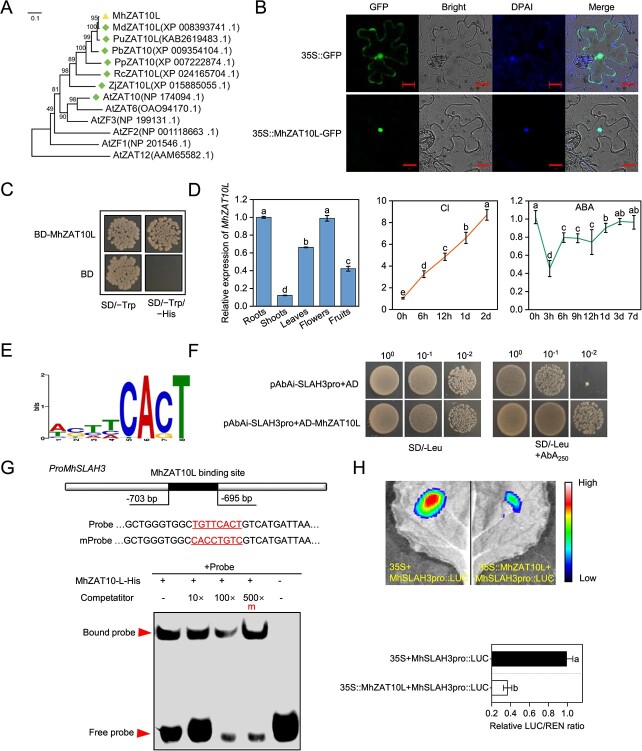
The characteristics, expression patterns, and interaction of MhZAT10L with the *MhSLAH3* promoter. **A** Phylogenetic analysis was conducted to compare MhZAT10L with its homologs from various plant species, including MdZAT10L (apple), PuZAT10L and PbZAT10 (pear), PpZAT10 (peach), RcZAT10 (rose), ZjZAT10L (maize), and AtZAT10, AtZAT6, AtZF3, AtZF2, AtZF1, and AtZAT12 (Arabidopsis). **B** Subcellular localization of MhZAT10L. DAPI dye was employed to label the nuclei. Bars, 20 μm. **C** Trans-transcriptional activity was assessed by plating the ‘Y2H Gold’ yeast strain carrying either the pGBKT7 (BD) empty vector or the BD-MhZAT10L fusion vector on selective media lacking Trp (SD/−Trp) or lacking both Trp and His (SD/−Trp/–His) for 3 days at 28°C. **D**  *MhZAT10L* relative expression level in different tissues, under 50 mM chloride (Cl) treatment, or under 10 μM abscisic acid (ABA) treatment. **E** The potential binding motif logo of ZAT10 was obtained from plantPAN v3.0. **F** Yeast one-hybrid (Y1H) assay between MhZAT10L and the *MhSLAH3* promoter. **G** Electrophoretic mobility shift assay (EMSA). The wild-type probe is a fragment of the *MhSLAH3* promoter fragment that contains a potential binding site (TGTTCACT) for MhZAT10L. The putative binding site ‘TGTTCACT’ was substituted with ‘CACCTGTC’ in the mutant probe. The presence or absence of corresponding proteins or competitors is indicated by the symbols ‘−’ and ‘+’. The presence of mutant probes is denoted by ‘m’. Competitors are designated as ‘10×, 100×, and 500×’. **H** Dual-luciferase reporter assay (DLR). The values represent the ratio of luciferase (LUC) activity to Renilla (REN) activity in tobacco leaves transiently expressing the genes. The data are shown as the mean ± standard deviation (*n* = 3 in D, *n* = 6 in H). Statistically significant differences (*P* < 0.05) are indicated by different letters in each column (one-way ANOVA).

To explore the regulation effect of ABA on *MhSLAH3*, we employed the promoter region (2000 bp upstream of ATG) of *MhSLAH3* (Table S4, see online supplementary material) as bait to screen a cDNA library using the yeast one-hybrid (Y1H) method (Table S1, see online supplementary material), and a homolog of C2H2 TF ZAT10, MhZAT10L, was obtained (Table S5, see online supplementary material; Fig. 3A).

The MhZAT10L was a nucleus-located protein (Fig. 3B) and had transcriptional activity (Fig. 3C). It had highest expression level in the roots and was strongly induced by Cl stress; however, it was inhibited by ABA, particularly within 1 day of treatment (Fig. 3D).

To further clarify the binding sites of MhZAT10L with the *MhSLAH3* promoter, we scanned the promoter using plantPAN version 3.0 and discovered a possible binding motif (TGTTCACT) of MhZAT10L (Fig. 3E). The Y1H assay validated the *in vitro* interaction between MhZAT10L and the *MhSLAH3* promoter (Fig. 3F). The probe and its equivalent mutation counterpart were labeled using biotin to carry out an electrophoretic mobility shift assay (EMSA). We observed an interaction between MhZAT10L and the *MhSLAH3* promoter by co-incubating the purified MhZAT10L-His fusion protein and the labeled probes (Fig. 3G). The formation of the DNA–protein complex and its brightness remained unaffected by the high concentrations of the mutant competitor probe (Fig. 3G). A dual-luciferase reporter (DLR) assay revealed that MhZAT10L repressed *MhSLAH3* expression (Fig. 3H). These findings suggest that MhZAT10L directly represses *MhSLAH3* transcription by binding to the ‘TGTTCACT’ motif.

**Figure 4:**
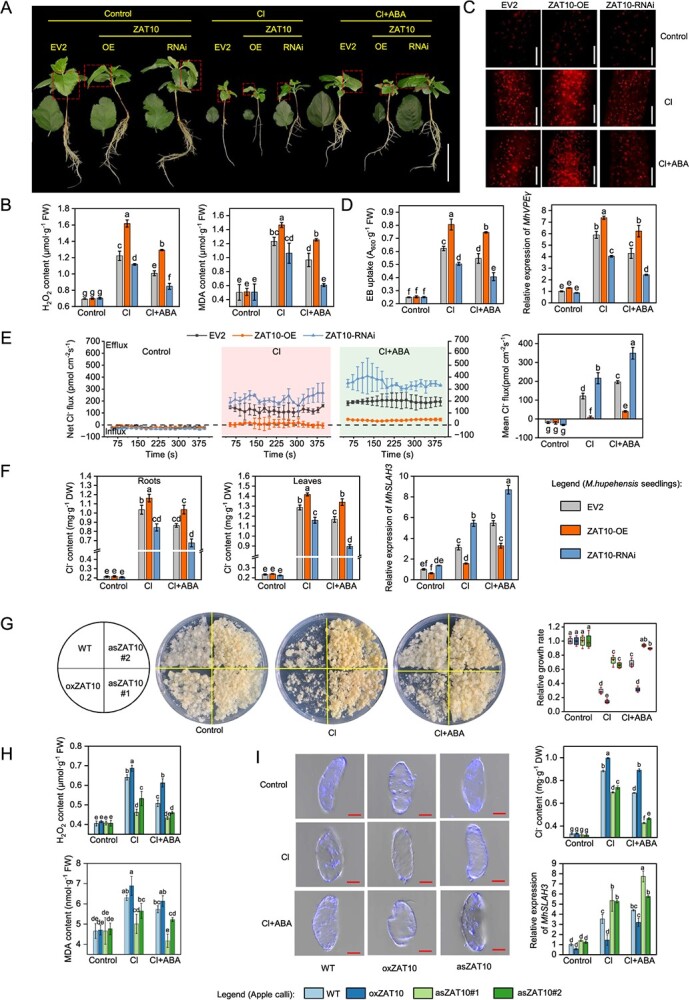
Effects of abscisic acid on cell death and net Cl^−^ flux in *MhZAT10L* transgenic hairy roots of *Malus hupehensis* and apple calli under chloride stress. **A** Phenotypic comparison between *MhZAT10L*-overexpressing (ZAT10-OE), empty expression vector (EV2), and *MhZAT10L*-suppressing (ZAT10-RNAi) hairy root *M. hupehensis* seedlings under 50 mM chloride (Cl) and 50 mM Cl with 10 μM abscisic acid (ABA) application. Bars, 5 cm. The phenotypic data are from one representative line out of at least three transgenic lines of ZAT10-OE or ZAT10-RNAi. **B** H_2_O_2_ and MDA content. **C** Distribution of dead cells from ZAT10-OE, EV2, and ZAT10-RNAi roots under 50 mM Cl and 50 mM Cl with 10 μM ABA application. Bars, 100 μm. **D** Cell death rate, relative expression level of *MhVPEγ*. **E** Net Cl^−^ flux. **F** Cl^−^ content in roots and leaves and the *MhSLAH3* relative expression level in ZAT10-OE, EV1, and ZAT10-RNAi roots under 50 mM Cl and 50 mM Cl with 10 μM ABA application. The data are represented by the means of transgenic lines ± standard deviation (*n* = 3). **G** Phenotypic comparison and relative growth rate of wild-type (WT) and *MhZAT10L* transgenic apple calli (oxZAT10 and asZAT10) under 150 mM Cl and 150 mM Cl with 10 μM ABA addition. **H** H_2_O_2_ and MDA content. **I** Distribution of Cl^−^ as indicated by the Cl^−^ reverse indicator MQAE, Cl^−^ content, and the relative *MdSLAH3* expression of WT, oxZAT10, and asZAT10 under 150 mM Cl and 150 mM Cl with 10 μM ABA addition. The data are donated as the mean ± standard deviation (except *n* = 4 in G, *n* = 3 in others). Statistically significant differences (*P* < 0.05) are indicated by different letters in each column (one-way ANOVA).

We then obtained multiple *M. hupehensis* seedlings that contained *MhZAT10L*-overexpressing (ZAT10-OE), *MhZAT10L*-suppressing (ZAT10-RNAi), and empty vector-expressing (EV2) hairy roots (Figs S4 and S5, see online supplementary material). The seedlings grew similarly under normal conditions (Fig. 4A). Under Cl stress, the growth inhibition of ZAT10-OE lines was more severe than others, whereas the ZAT10-RNAi lines maintained relatively healthy growth (Fig. 4A). The H_2_O_2_ and MDA content were highest in the ZAT10-OE roots, whereas the ZAT10-RNAi lines had the lowest content (Fig. 4B). Brighter PI fluorescence and a higher EB uptake value and *MhVPEγ* expression level were observed in ZAT10-OE roots. In contrast, the ZAT10-RNAi roots exhibited the opposite result (Fig. 4C), suggesting that silencing *MhZAT10L* resulted in a reduction in cell death. The net Cl^−^ flux of the EV2, ZAT10-OE, and ZAT10-RNAi lines showed efflux from roots under Cl stress. Nevertheless, the ZAT10-OE lines had the lowest Cl^−^ efflux rate and the highest Cl^−^ content, while the ZAT10-RNAi lines had the highest Cl^−^ efflux rate and the lowest Cl^−^ content (Fig. 4D and E). In addition, the expression level of *MhSLAH3* was suppressed in the ZAT10-OE lines but elevated in the ZAT10-RNAi lines under Cl stress (Fig. 4F).

ABA enhanced the growth of Cl-stressed *MhZAT10L* transgenic *M. hupehensis* by ameliorating growth inhibition, reducing the H_2_O_2_ and MDA content, and decreasing the cell death rate. It also resulted in a comparatively higher rate of Cl^−^ efflux and elevated *MhSLAH3* expression, while reducing the Cl^−^ content (Fig. 4A–F). We noted that the effect of ABA on the ZAT10-OE lines was less significant than its effect on ZAT10-RNAi and EV2 lines (Fig. 4A–F). In other words, the beneficial effects of ABA were mitigated by *MhZAT10L* overexpression.

One *MhZAT10L*-overexpressing (oxZAT10) and two *MhZAT10L*-suppressing (asZAT10#1, #2) apple calli were also obtained (Fig. S6, see online supplementary material). asZAT10 grew better under Cl stress (Fig. 4G–I), had a lower H_2_O_2_ and MDA content, lower Cl^−^ accumulation as indicated by the brighter fluorescence of MQAE (the Cl^−^ reverse indicator), lower Cl^−^ content, and higher *MhSLAH3* expression (Fig. 4H and I). oxZAT10 grew worse and had a higher Cl^−^ accumulation (Fig. 4G–I). Similar to transgenic *M. hupehensis*, ABA improved the poor growth of Cl-stressed apple calli, but the effect of ABA was counteracted by MhZAT10L.

### MhABI5 elevates the Cl^−^ efflux rate by regulating *MhZAT10L* expression

**Figure 5:**
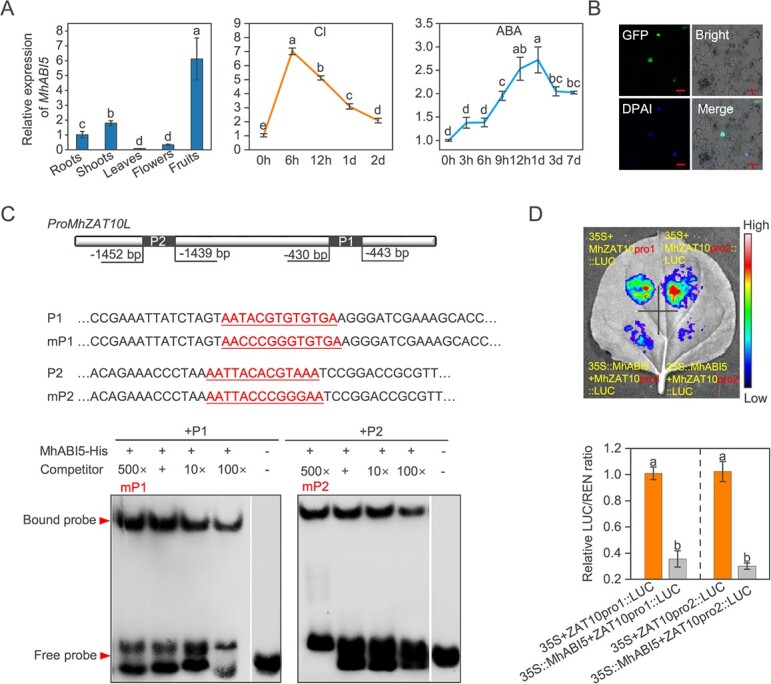
Characteristics, expression patterns, and interaction of MhABI5 with the *MhZAT10L* promoter. **A** Relative expression level of *MhABI5* in different tissues, under 50 mM chloride (Cl) treatment, and under 10 μM abscisic acid (ABA) treatment. **B** Subcellular localization of MhABI5. DAPI was utilized for nuclear labeling. Bar, 20 μm. **C** Electrophoretic mobility shift assay (EMSA). Two probes (with the highest similar score) containing the presumed MhABI5 binding sites, AATACGTGTGTGA and AATTACACGTAAA, were used. Mutant probes with mutated binding sites, named mP1 (AACCCGGGTGTGA) and mP2 (AATTACCCGGGAA), were also designed. **D** The transient expression assay demonstrated that MhABI5 suppresses *MhZAT10L* expression. The data are shown as the mean ± standard deviation (*n* = 3 in A, *n* = 6 in D). Statistically significant differences (*P* < 0.05) are indicated by different letters in each column (one-way ANOVA).

We scanned the *MhZAT10L* promoter(Table S6, see online supplementary material) in order to delve deeper into the regulatory effects of ABA on MhZAT10L, and we found 14 potential binding sites of MhABI5 (Tables S8 and S9, see online supplementary material). *MhABI5* (Table S7, see online supplementary material) is highly expressed in fruits, shoots, and roots, and it was also upregulated by Cl or ABA (Fig. 5A). It localized in the nucleus (Fig. 5B). The EMSA assay demonstrated the interaction between MhABI5 and its two binding sites with the most potential (i.e., those with the highest similarity score) on the *MhZAT10L* promoter (Fig. 5C; Table S9, see online supplementary material), and the DLR assay indicated that MhABI5 suppressed *MhZAT10L* expression (Fig. 5D).

**Figure 6:**
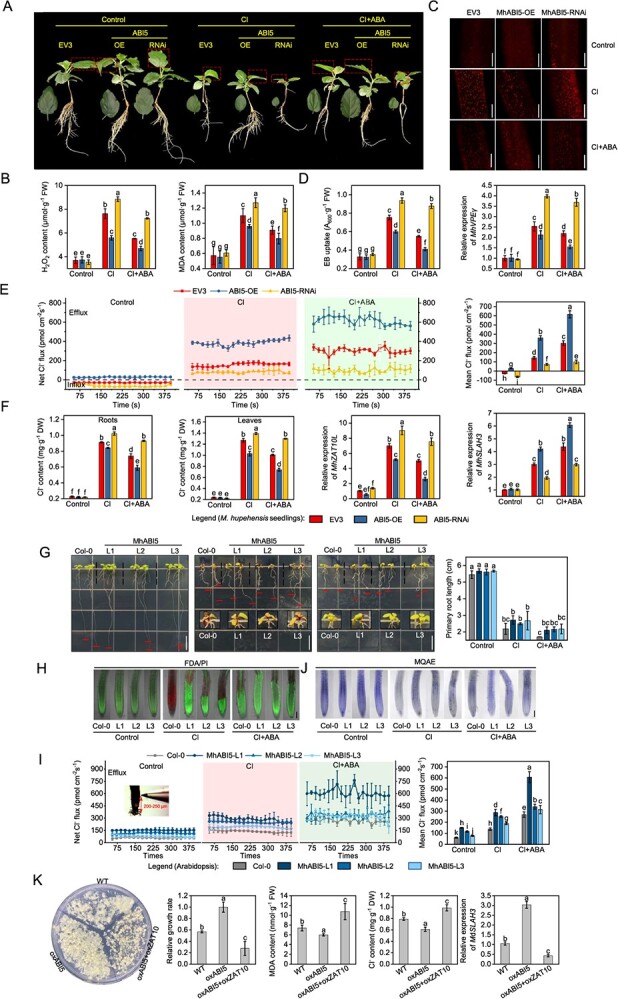
Effect of abscisic acid on cell death and net Cl^−^ flux in *MhABI5* transgenic plants under chloride stress. **A** Phenotypic comparison between *MhABI5*-overexpressing (ABI5-OE), empty expression vector (EV3), and *MhABI5*-suppressing (ABI5-RNAi) hairy root *Malus hupehensis* seedlings under 50 mM chloride (Cl) and 50 mM Cl with 10 μM abscisic acid (ABA) application. Bars, 5 cm. The phenotypic data are from one representative line out of at least three transgenic lines of ABI5-OE or ABI5-RNAi. **B** H_2_O_2_ and MDA content. **C** Distribution of dead cells in ABI5-OE, EV3, and ABI5-RNAi roots under 50 mM Cl and 50 mM Cl with 10 μM ABA application. Bars, 100 μm. **D** Cell death rate, *MhVPEγ* relative expression. **E** Net Cl^−^ flux, **F** Cl^−^ content, and relative expression levels of *MhZAT10L* in ABI5-OE, EV3, and ABI5-RNAi roots under 50 mM Cl and 50 mM Cl with 10 μM ABA application. The data are donated by the mean of transgenic lines ± standard deviation (*n* = 3). **G** Phenotypic comparison between Col-0 and Arabidopsis lines heterologously expressing *MhABI5* (L1–L3) under 150 mM Cl and 150 mM Cl with 1 μM ABA application. Bars, 1 cm. **H** Cell death indicated by FDA/PI double stain of Col-0 and L1–L3 roots under 150 mM Cl and 150 mM Cl with 1 μM ABA application. Bars, 100 μm. **I** Net Cl^−^ flux and **J** Cl^−^ distribution indicated by the MQAE of ABI5-OE, EV3, and ABI5-RNAi roots under 150 mM Cl and 150 mM Cl with 1 μM ABA application. Bars, 100 μm. **K** Phenotypic comparison, relative growth rate, Cl^−^ content, and *MdSLAH3* expression level between WT, *MhABI5*-overexpressing, as well as *MhABI5* and *MhZAT10L* co-overexpressing apple calli under 150 mM Cl. The data are shown as the mean ± standard deviation (*n* = 3). Statistically significant differences (*P* < 0.05) are indicated by different letters in each column (one-way ANOVA).

Multiple *MhABI5*-overexpressing (ABI5-OE), *MhABI5*-suppressing (ABI5-RNAi), and empty-expression vector (EV3) transgenic hairy roots were generated in *M. hupehensis* (Figs S4 and S5, see online supplementary material). Their growth was similar without Cl treatment (Fig. 6A). However, the ABI5-OE lines exhibited a better growth than the EV3 lines under Cl stress, while the reverse phenotype was observed in the ABI5-RNAi lines (Fig. 6A). The H_2_O_2_ and MDA content in the ABI5-OE roots were the lowest, whereas those in the ABI5-RNAi roots were the highest (Fig. 6B). In addition, the ABI5-OE roots exhibited a darker PI fluorescence, lower EB uptake value, and decreased *MhVPEγ* expression, while the ABI5-RNAi roots showed the opposite results (Fig. 6C and D). ABI5-OE lines exhibited the highest rate of Cl^−^ efflux from roots and the lowest Cl^−^ content in roots and leaves. Furthermore, it showed the highest expression of *MhSLAH3* and the lowest expression of *MhZAT10L*. In contrast, the ABI5-RNAi lines demonstrated opposite expression patterns for *MhSLAH3* and *MhZAT10L* (Fig. 6E and F).

After ABA addition, there was a significant improvement in seedling growth and a reduction in cell death (Fig. 6A and B). It also reduced Cl^−^ accumulation and *MhZAT10L* expression but increased *MhSLAH3* expression (Fig. 6E and F); however, these effects were significantly weaker in ABI5-RNAi than in ABI5-OE and EV3 (Fig. 6A–F). This suggests that silencing MhABI5 moderated the ABA function on Cl^−^ efflux by elevating *MhZAT10L* expression.

We also generated three Arabidopsis lines heterologously expressing *MhABI5* (L1–L3) (Fig. S7, see online supplementary material). Compared to Col-0, *MhABI5* expression resulted in better growth, longer primary roots in Arabidopsis under Cl stress (Fig. 6G), and reduced root cell death, as indicated by FDA/PI fluorescence (Fig. 6H). In addition, the Cl^−^ efflux rate was also elevated, and the Cl^−^ content indicated by MQAE was also correspondingly decreased (Fig. 6I). Except for the inhibited primary length, ABA addition resulted in higher Cl^−^ efflux and lower Cl^−^ content and cell death in Arabidopsis heterologously expressing *MhABI5* (Fig. 6G–I), indicating that ABA enhances MhABI5 function in resisting Cl stress.

Apple calli overexpressing *MhABI5* (oxABI5) and those co-overexpressing *MhABI5* and *MhZAT10L* (oxABI5 + oxZAT10) were also obtained (Fig. S8, see online supplementary material). The oxABI5 had a better growth, lower Cl^−^ content, lower MDA content, and higher *MdSLAH3* expression levels than WT under Cl stress; however, the oxABI5 + oxZAT10 apple calli had a worse growth and higher Cl^−^ content than WT (Fig. 6K), suggesting that *MhZAT10L* reverses the effects of *MhABI5* under Cl stress.

## Discussion

Exogenous ABA inhibits Cl^−^ absorption [24]. We found that exogenous ABA significantly increased Cl^−^ efflux from roots and subsequently decreased Cl^−^ accumulation (Fig. 1). The reduction in endogenous ABA levels results in less Cl^−^ exclusion from the root apex [25]. Our prior report also evidenced that improving endogenous ABA reduces Cl^−^ accumulation and results in better plant growth after Cl^−^ stress [13]. Here, exogenous ABA also improved the growth of Cl-stressed *M. hupehensis* by reducing H_2_O_2_ generation and alleviating cell damage and death (Fig. 1). Thus, ABA probably plays a pivotal role in the adaptation of *M. hupehensis* to Cl stress.

The Cl^−^ export in plants was partially regulated by some Cl^−^ channels, such as ALMT9, SLAC/SLAH, and CLCs, in which SLAC/SLAH exhibit more pivotal roles in regulating Cl^−^ efflux than other channels [15, 40]. There were 4 SLAC/SLAH homologs in the apple proteome, namely, MdSLAC1 and MdSLAH1 to 3 (Fig. S2A, see online supplementary material). Only the *MdSLAH3* homolog in *M. hupehensis* (*MhSLAH3*) was strongly expressed in roots (Fig. S2B, see online supplementary material) and obviously induced by Cl and ABA (Fig. 2A). *MhSLAH3* suppression in roots weakened the Cl^−^ exclusion from the roots, but its overexpression elevated the Cl^−^ efflux rate and reduced Cl^−^ accumulation, thereby alleviating membrane damage and cell death in roots (Fig. 2D–H), indicating that *MhSLAH3* negatively regulates Cl^−^ accumulation and damage by promoting Cl^−^ efflux from roots. In addition, all of the *MhSLAH3* transgenic seedlings exhibited improved growth, a higher Cl^−^ efflux rate, and lower Cl^−^ accumulation, as well as reduced MDA and H_2_O_2_ content and cell death following the addition of exogenous ABA (Fig. 2D–H). ABA also modulates transpirational pull by controlling stomatal movement, which influences the uptake and transport of some nutrient ions. As a type of nutrient ion, the import and transportation of Cl^−^ is also influenced by ABA-modulated transpirational pull [41, 42]. However, SLAH3-mediated Cl^−^ efflux should not be ignored. We found that silencing *MhSLAH3* in *M. hupehensis* roots greatly weakened the positive effects of ABA, such as elevating the Cl^−^ efflux rate and alleviating cell death (Fig. 2D–H). Overall, the promotion of ABA on Cl^−^ efflux during Cl stress is achieved through MhSLAH3.

Further, a ZAT10 homolog (MhZAT10L) was identified as a transcriptional repressor of *MhSLAH3* (Fig. 3). *MhZAT10L* was highly expressed in the roots, and its transcription was strongly induced by Cl but significantly inhibited by ABA (Fig. 3D). *MhZAT10L* suppression enhanced the Cl^−^ efflux rate from roots, but its overexpression reduced the Cl^−^ efflux rate from roots and decreased the Cl^−^ accumulation. Thus, the H_2_O_2_ and MDA content was diminished, and cell death was alleviated in *M. hupehensis*. Similar results were also obtained in apple calli (Fig. 4). These findings suggest that MhZAT10L plays an opposite role to MhSLAH3 during Cl stress (Figs 2 and 4). Prior studies have shown that SLAC/SLAH is regulated by different kinases [43, 44], but limited studies have focused on the regulation of its transcriptional level. We found that MhZAT10L could bind to *MhSLAH3* promoter and activate its transcription (Fig. 3). *MhSLAH3* expression was also elevated by *MhZAT10L* suppression but inhibited by *MhZAT10L* overexpression under Cl stress (Fig. 4F). Thus, the mitigation of MhZAT10L on Cl^−^ efflux rate is achieved partly depending on the repression of *MhSLAH3* transcription. Intriguingly, ABA elevated Cl^−^ efflux and alleviated cell death in *MhZAT10L* transgenic plants. However, the impacts of ABA were counteracted by *MhZAT10L* overexpression, as *MhZAT10L*-overexpressing plants displayed a lower roots Cl^−^ efflux rate and higher Cl^−^ accumulation than *MhZAT10L*-suppressing and EV(WT) plants after ABA addition (Fig. 4). These findings indicate that ABA modulates Cl^−^ efflux by regulating *MhZAT10L* transcription.

ABA signaling is essential for ABA’s physiological effects, and ABI5 is regarded as a crucial TF in ABA signaling that mediates ABA-regulated stress responses [26]. Here, we searched 14 potential binding sites of ABI5 at the *MhZAT10L* promoter (Tables S7 and S8, see online supplementary material) and isolated the homolog of ABI5 from *M. hupehensis* (MhABI5). MhABI5 was highly expressed in roots, and it was a nucleus-located protein that simultaneously strongly responded to Cl and ABA (Fig. 5A and B). *MhABI5* overexpression greatly accelerated the Cl^−^ efflux rate from roots and reduced Cl^−^ accumulation, but its suppression decreased the Cl^−^ efflux rate and increased Cl^−^ accumulation, the generation of H_2_O_2_ and MDA, and cell death during Cl stress (Fig. 6). Therefore, MhABI5 negatively modulates Cl^−^ accumulation and damage in Cl-stressed *M. hupehensis*. Further, MhABI5 bound to *MhZAT10L* promoter and repressed *MhZAT10L* expression (Fig. 5). *MhABI5* overexpression caused *MhZAT10L* transcription to a lower level, thereby elevating *MhSLAH3* expression (Fig. 6F), and its effects on Cl^−^ content were reversed by *MhZAT10L* (Fig. 6K). Consistent with MhABI5, ABA also elevated the Cl^−^ efflux rate and alleviated cell death (Fig. 1); however, the ABA function was greatly weakened by *MhABI5* silencing (Fig. 6). Overall, ABA regulates MhZAT10L-mediated Cl^−^ efflux through MhABI5.

**Figure 7:**
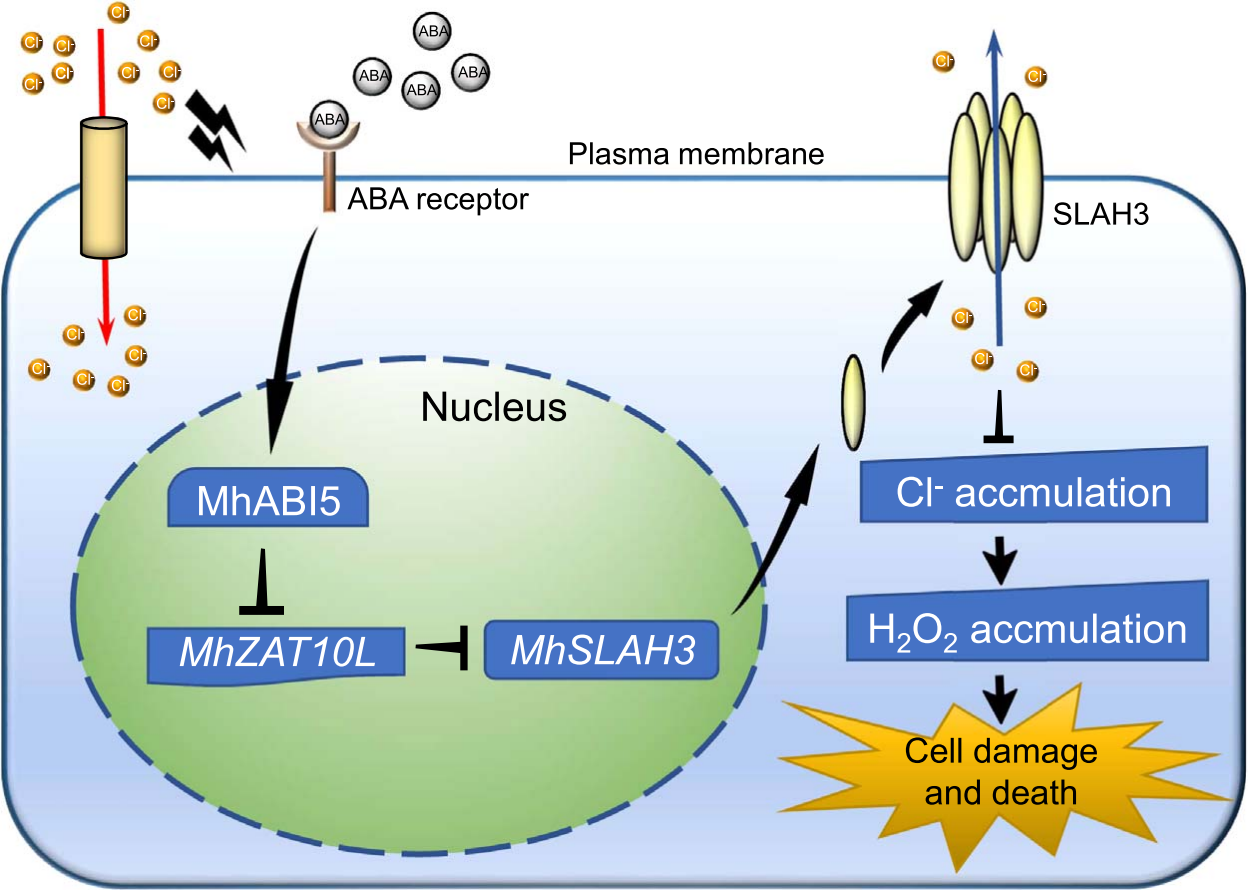
A working model showing that the ABI5-ZAT10-SLAH3 transcription module mediates ABA to regulate Cl^−^ accumulation and damage under chloride stress. During chloride stress, ABA activates MhABI5, and activated MhABI5 binds to the *MhZAT10L* promoter and represses its transcription; repressed MhZAT10L releases *MhSLAH3* to accelerate the Cl^−^ efflux from roots and subsequently alleviates Cl^−^ overaccumulation, thereby decreasing H_2_O_2_ accumulation. Therefore, cell damage and death are alleviated. ABA: abscisic acid.

Taken together, we propose a working model to summarize our findings (Fig. 7). The application of exogenous ABA activates MhABI5 during Cl stress. Activated MhABI5 binds to the *MhZAT10L* promoter and suppresses *MhZAT10L* transcription. Repressed MhZAT10L further releases *MhSLAH3* to accelerate Cl^−^ efflux and subsequently alleviates Cl^−^ accumulation, ultimately decreasing the generation of H_2_O_2_ and H_2_O_2_-caused cell damage and death. Therefore, the ABI5-ZAT10-SLAH3 module is an effective pathway for ABA-regulated adaptation in plants to Cl stress, particularly in Cl-sensitive horticultural plants. Our findings establish a theoretical foundation for augmenting the genetic enhancement of apple rootstock’s salt tolerance, and could also facilitate the development and utilization of saline-alkali land.

## Materials and methods

### Plant materials and growth conditions

Materials, including *M. hupehensis* seedlings, ‘Orin’ apple calli, Arabidopsis (ecotype Columbia), and tobacco (*Nicotiana benthamiana*) were utilized in this study. The cultivation of *M. hupehensis* seedlings, ‘Orin’ apple calli, and tobacco were carried out as described previously [39]. For *M. hupehensis* seedlings, the nutrient solution was replaced every five days. Arabidopsis were cultured as described previously [17].

The tissue expression patterns of genes were analysed following the methodology described by Song *et al.* [39].

### Treatments of wild-type plants

Given that the primary forms of Cl^−^ in the soil are NaCl, KCl, CaCl_2_, and MgCl_2_, and considering the detrimental effects of Na^+^, a 50 mM Cl solution—that is, ½ Hoagland’s solution containing 0 mM NaCl, 25 mM KCl, 6.125 mM CaCl_2_, and 6.125 mM MgCl_2_ [45]—was utilized to treat *M. hupehensis* seedlings. Roots were collected at 0 h, 6 h, 12 h, 1 d, and 2 d. For ABA treatment, the seedlings were placed in ½ Hoagland’s solution containing 10 μM ABA, and roots were sampled at 0 h, 3 h, 6 h, 9 h, 12 h, 1 d, 3 d, and 7 d. Three biological replicates were implemented for each treatment. All collected samples were immediately frozen in liquid nitrogen and preserved at a temperature of −80°C.

*M. hupehensis* seedlings at the three- or four-leaf stage and exhibiting similar growth were divided into six experimental groups, each containing at least 10 plants per group. The seedlings were then exposed to different treatments: ½ Hoagland’s solution (control group), 50 mM Cl (Cl group), or 50 mM Cl supplemented with 5, 10, 20, or 30 μM ABA (Cl + ABA group). After a 30-day treatment period, their phenotypes were documented.

Fifteen-day-old apple calli lines were cultured on MS medium alone, MS medium containing 150 mM Cl, and MS medium containing 150 mM Cl supplemented with 5, 10, 20, or 30 μM ABA. Following 20-day growth, the phenotypes were documented.

Four-day-old Arabidopsis were transferred to ½ MS medium alone or supplemented with 150 mM Cl, and 0.1, 0.5, 1.0, 2.0, or 4.0 μM ABA. The observation and documentation of the phenotypes were carried out after a 7-day growth period.

### Total RNA extraction and quantitative real-time PCR analysis

Total RNA extraction and quantitative real-time PCR (qRT-PCR) analysis was carried out using a method described by Song *et al.* [17]. The primers utilized in the analysis were documented in Table S2 (see online supplementary material). The study encompassed at least nine replicates, comprising three technical replicates and three biological replicates.

### Identification, isolation, and bioinformatics analysis of genes

SLAC/SLAH members in the apple proteome (GDDH13 version 1.1) were identified following our prior method [17]. All of the genes in the study were isolated from the roots of *M. hupehensis* using Phanta Max Super-Fidelity DNA Polymerase (Vazyme, Nanjing, China). Phylogenetic analysis was conducted utilizing MEGA version 7.0, and multiple sequence alignment was carried out utilizing Muscle [46]. Transmembrane prediction was conducted utilizing TMHMM 2.0 (https://services.healthtech.dtu.dk/services/TMHMM-2.0/).

### Subcellular localization and transcription activity analysis

To visualize the subcellular localization, *MhSLAH3*, *MhZAT10L*, and *MhABI5* were inserted into pBI121-GFP vector and linked upstream of the GFP under the control of the CaMV 35S to create *35S::MhSLAH3-GFP*, *35S::MhZAT10L-GFP*, and *35S::MhABI5-GFP* constructs, respectively. They were transiently expressed in tobacco leaves through the mediation of *Agrobacterium* GV3101 following a pervious protocol [47]. After a 2-day incubation period, the tobacco leaves were observed using a laser confocal microscope (LSM880 Zeiss, Jena, Germany). DAPI and StREM1.3 [48] were used as nuclear and plasma membrane markers, respectively. The excitation and emission wavelengths for GFP, YFP, and DAPI were 488, 514, and 380 nm, and 495–515, 535–590, and 430–450 nm, respectively.

To investigate the transcription activity of MhZAT10L, its cDNA was cloned into the pDEST-GKBT7 vector and transformed into ‘Y2H Gold’ yeast. The yeast was cultured on SD/−Trp and SD/−Trp/–His media, and growth was assessed after 3 days at 28°C.

### Genetic transformation and molecular confirmation of transgenic plants

The *MhSLAH3*, *MhZAT10L*, and *MhABI5*, along with their respective reverse specific sequences of approximately 200–300 bp (to suppress endogenous gene expression), were cloned into pGWB405 vector (with a kanamycin marker) to fuse the *EGFP* gene and under the control of 35S promoter.

The genetic transformation and identification of *M. hupehensis* hairy roots were performed following the method reported by Song *et al.* [39]. The fusion expression vectors, including *pGWB405-MhZAT10L*, *pGWB405-antiMhZAT10L*, and *35S::MhABI5-GFP* (with a hygromycin marker) were utilized to produce transgenic apple calli using a previously described method [49]. Fusion vector *35S::MhABI5-GFP* was introduced into Arabidopsis following the method described in our prior study [50].

### Treatments of transgenic plants

Every transformed *M. hupehensis* line was cultured for approximately 40 days and then divided into three groups, with at least 10 plants in every group. Subsequently, they were transferred to the control, Cl, and Cl + ABA (10 μM) groups and treated for 30 days. Their phenotypes were documented and counted. The roots were sampled to determine physiological indices and gene expression.

Each apple calli line was plated on MS medium, MS medium containing a 150 mM Cl, and MS medium containing a 150 mM Cl and 10 μM ABA. Following a 20-day growth period, the growth phenotypes, MDA content, Cl^−^ content, and *MdSLAH3* expression were determined.

Four-day-old Arabidopsis lines were transferred into normal media, media supplemented with 150 mM Cl, and media supplemented with 150 mM Cl and 1 μM ABA. Following a treatment period of 7 days, the root phenotypes were quantified and recorded. The cell death and disruption of Cl^−^ in the roots were also assessed.

### Yeast one-hybrid assays

Y1H assay was carried out follow the pervious description [39]. In brief, the promoters of *MhSLAH3* and *MhZAT10L* were analysed using plantPAN v3.0 to find binding motifs of MhZAT10L and MhABI5, respectively [51]. The binding sequences were cloned into the pAbAi vector, after which the Y1H assay was performed according to the manufacturer’s guidelines.

### Electrophoretic mobility shift assays

The introduction and prurition MhZAT10L-His and MhABI5-His fusion proteins were carried out as described previously [39]. Then the probes labeled by biotin and fusion proteins were incubated for 30 min at 24°C. The cold-competition was performed using the unlabeled probes.

### Dual-luciferase reporter assays

Promoter fragments containing the core binding sequence of *MhSLAH3* and *MhZAT10L* were inserted into pGreen II 0800-LUC to generate *MhSLAH3pro::LUC* and *MhZAT10Lpro::LUC* fusion vectors (Reporter). Additionally, the *MhZAT10L* and *MhABI5* were inserted into pGreen II 62-SK to create fusion vectors *35S::MhZAT10L* and *35S::MhABI5* (Effector). Subsequently, the vectors were transiently transformed into tobacco leaves, as described by He *et al.* [32]. After culturing for 3 days, luciferase expression was determined using an *in vivo* imaging system (Xenogen, Sunnyvale, CA, USA), and the LUC/REN ratio was determined using a Dual-Luciferase Reporter Assay Kit (Beyotime Biotechnology, Shanghai, China).

### Physiological index assay

The MDA content, H_2_O_2_ content, and cell death were assayed as described prior [17].

Net Cl^−^ flux in *M. hupehensis* and Arabidopsis roots was measured using NMT, following a previously published description with minor adjustments [49]. Briefly, the microsensors of Cl^−^ were calibrated using different concentrations of Cl^−^ solution (i.e., 2 and 0.2 mM) before measurement. The roots (1.5 cm) of *M. hupehensis* or Arabidopsis with root tips were placed in the measuring solution [0.05 mM KCl, 0.05 mM CaCl_2_, 0.05 mM MgCl_2_, 0.25 mM NaCl, 0.3 mM HEPES, 0.2 mM Na_2_SO_4_, pH 6.0] to equilibrate for 10 min and subsequently tested for 7 min (discarding first-minute data in analysis). The net Cl^−^ flux in the root meristematic zone of *M. hupehensis* and Arabidopsis (200–250 μm from the root tip) was tested after 10 and 7 days of treatment, respectively. Every treatment was repeated at least three times.

The measurements of intracellular Cl^−^ using MQAE and Cl^−^ content in apple calli and Arabidopsis were determined as previously described [17].

### Statistical analysis

The data processing system (DPS) was utilized to analyse the experimental data, and Origin version 2023 (OriginLab Corporation, Northampton, MA, USA) was used to generate the figures. The significance of the indices in the different treatments and transgenic lines was determined using Duncan’s multiple range test (*P* < 0.05) and two-tailed Student’s *t*-test (**P < 0.01, *P < 0.05).

## Supplementary Material

Web_Material_uhae200

## Data Availability

All data are available in the main text or the supplementary information.
