# Supplementary material for: Abscisic acid regulates Cl− efflux via the ABI5-ZAT10-SLAH3 module in chloride-stressed Malus hupehensis
Source: Hortic Res. 2024 Jul 24;11(9):uhae200. doi: 10.1093/hr/uhae200 (PMC11387005; doi:10.1093/hr/uhae200)
Supplement: Web_Material_uhae200 [file web_material_uhae200.zip › Supplementary Figures--HR-R1 (clean)-5.15.docx]

**Supplementary Figures for**

**Abscisic acid regulates Cl^–^ efflux via the ABI5-ZAT10-SLAH3 module in chloride-stressed *Malus hupehensis***

Jianfei Song, Junhong Yan, Baozhen Sun, Bing Chen, Xiaoyue Zhu, Hongcai Wei, Zhilong Bao, Fangfang Ma, Weiwei Zhang*, Hongqiang Yang*

College of Horticulture Science and Engineering/Apple technology innovation center of Shandong Province, Shandong Agricultural University, Tai’an, 271018, Shandong, China

***Correspondence:**

Hongqiang Yang ([hqyang@sdau.edu.cn](mailto:hqyang@sdau.edu.cn)); Weiwei Zhang ([zhangww@sdau.edu.cn](mailto:Zhangww@sdau.edu.cn))

The PDF file includes:

Figure S1 to Figure S8


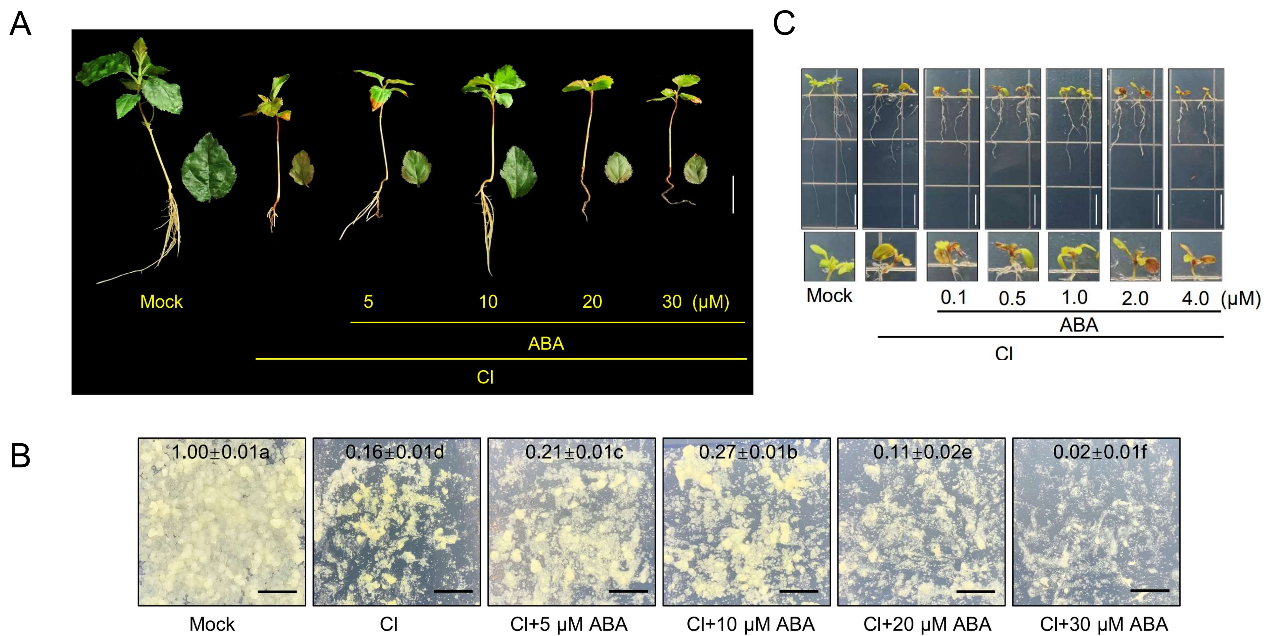


**Figure S1** **Effect of abscisic acid on the growth of chloride-stressed plants.**

(**A**) Phenotypic comparison of *M. hupehensis* seedlings under normal conditions (mock), 50 μM chloride (Cl), and 50 μM Cl supplemented with different concentrations of ABA. Scale bars = 2 cm. (**B**) Phenotypic comparison of apple calli under normal conditions, 150 μM Cl, and 50 μM Cl supplemented with different concentrations of ABA. Scale bars = 1 cm. (**C**) Phenotypic comparison of Arabidopsis under normal conditions, 150 μM Cl, and 150 μM Cl supplemented with different concentrations of ABA. Scale bars = 1 cm. The data are shown as the mean ± standard deviation (n = 3). Statistically significant differences (*P* < 0.05) are indicated by different letters in each column (one-way ANOVA).


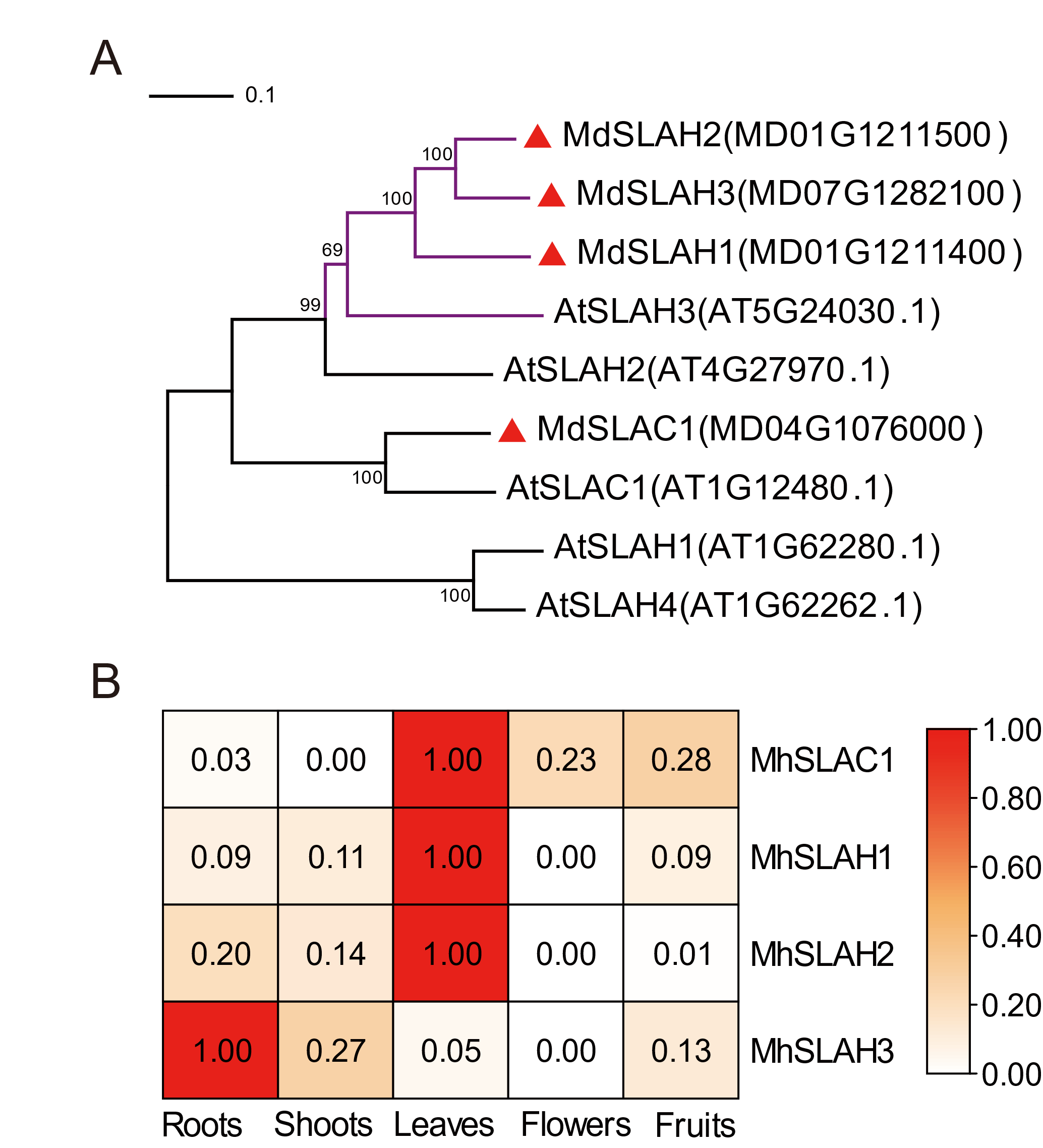


**Figure** **S2 Phylogenetic analysis and tissue expression patterns of *SLAC/SLAH* from apple.**

**(A)** Phylogenetic analysis of SLAC/SLAH from apple and Arabidopsis. Apple SLAC/SLAH members are labeled with red triangles. **(B)** Tissue expression patterns of *SLAC/SLAH* homologs in *Malus hupehensis*, which is commonly used as apple rootstock. The values were normalized using a zero-to-one method.


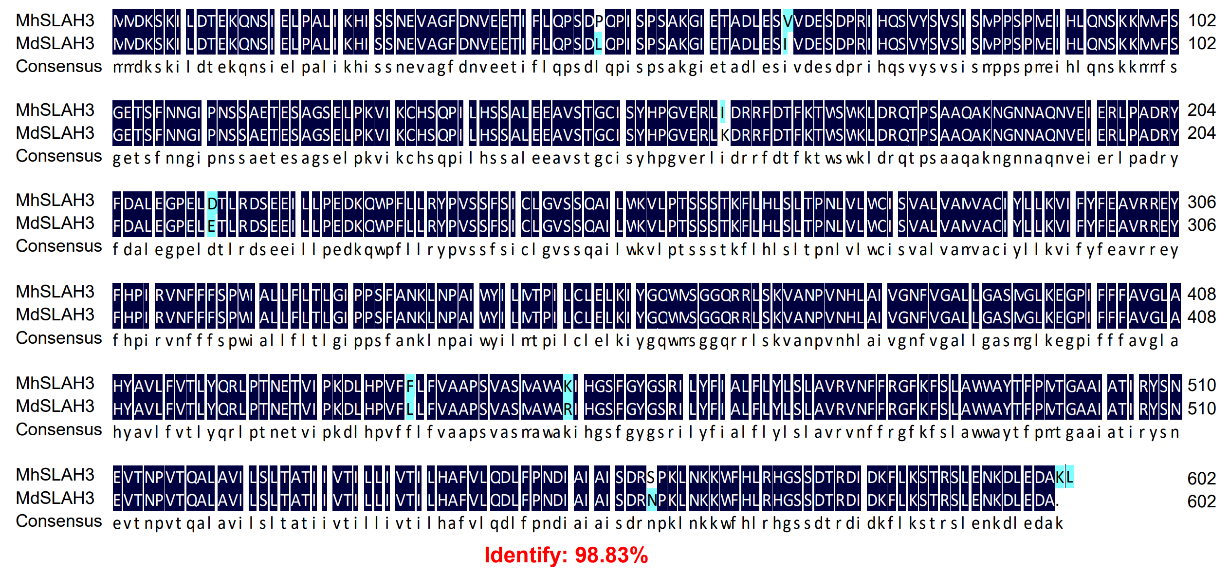


**Figure S3 Sequence alignment of SLAH3 from apple (MdSLAH3) and *Malus hupehensis* (MhSLAH3).**

The two sequences have a shared identity of 98.83%. The analysis was performed using DNAMAN version 2.0.


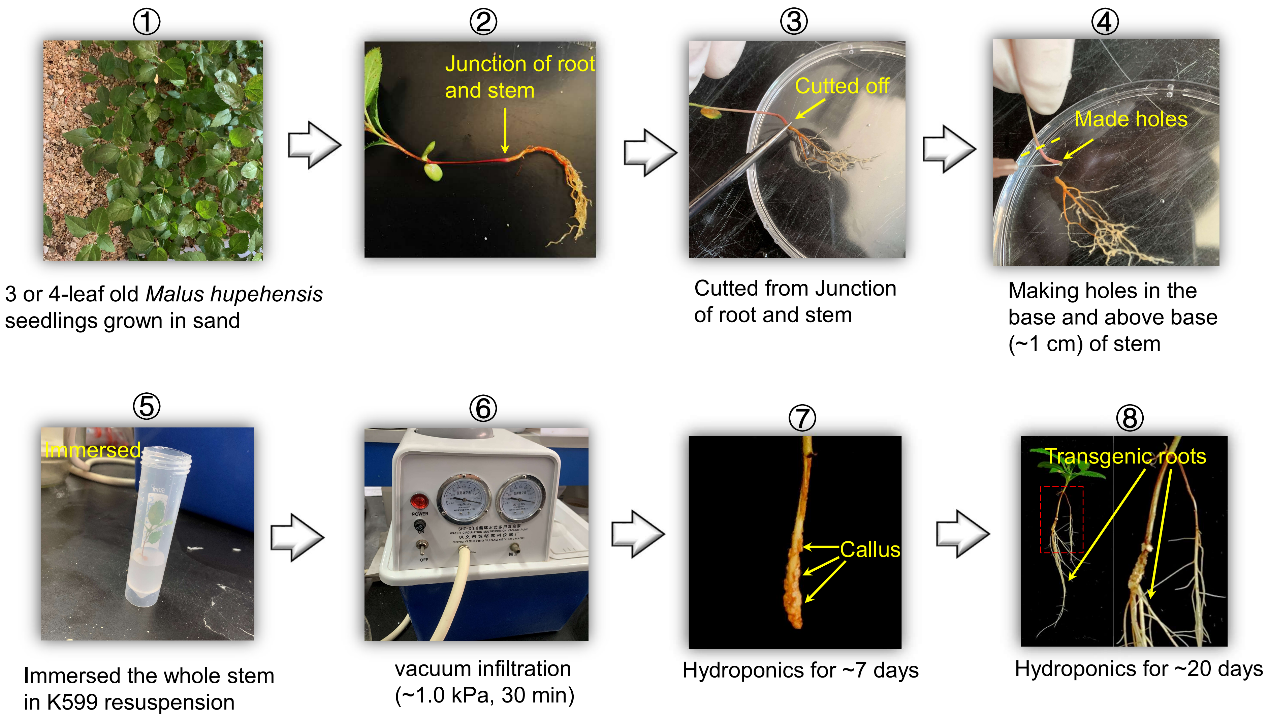


**Figure** **S4 Flow diagram of hairy root transformation mediated by *Agrobacterium rhizogenes* in *Malus hupehensis*.**

*Agrobacterium rhizogenes* K599 carrying fusion vectors was resuspended to an OD_600_ value of 1.0 using an infiltration solution containing 10 mM MgCl_2_, 10 mM 2-(N-morpholino) ethanesulfonic acid-KOH (MES-KOH, pH=5.2), and 100 μM acetosyringone. *Malus hupehensis* seedlings at the three- or four-leaf stage and grown in sand were cut from the root–stem junction, and holes were made in the shoot base. Subsequently, they were placed in the infiltration solution and vacuumed for 30 min, and then they were transferred to a ½ Hoagland’s solution for normal culturing. The callus and adventitious roots were observed after 7 and 20 days, respectively.


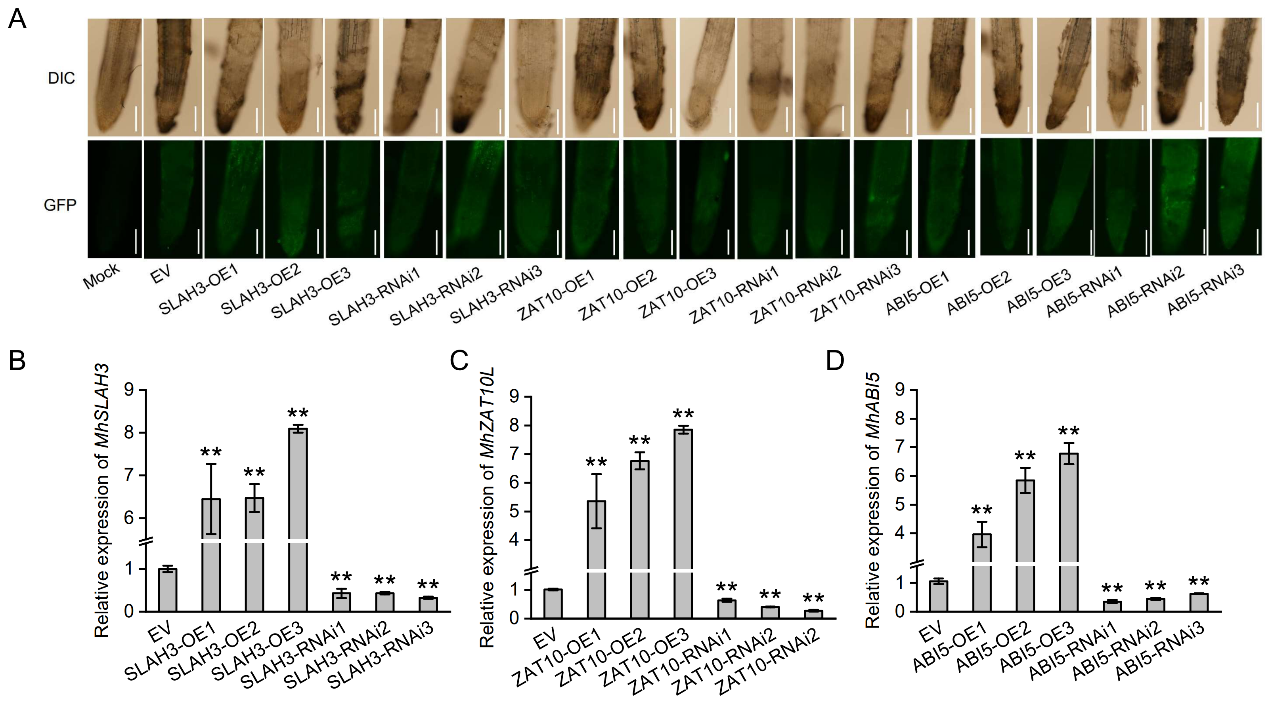


**Figure** **S5 Identification of transgenic hairy roots in *Malus hupehensis*.**

**(A)** GFP fluorescence analysis of transgenic hairy roots. The wild-type roots (mock) were used as a control to prevent the plant’s spontaneous fluorescence from the plant itself. Non-transgenic roots without GFP fluorescence were removed, and chimeric plants consisting of transgenic roots and wild-type shoots continued to grow in the ½ Hoagland's solution for 30 days. GFP, green fluorescent protein. Bright, sunny field. Bars, 200 µm. **(B)** Relative expression of *MhSLAH3* in EV and *MhSLAH3* transgenic hairy roots. **(C)** Relative expression of *MhZAT10L* in EV and *MhZAT10L* transgenic hairy roots. **(D)** Relative expression of *MhABI5* in EV and *MhABI5* transgenic hairy roots. The data are presented as the mean ± standard deviation (n = 3). All asterisks above the column denote significant differences detected by a two-tailed Student's *t*-test: ***P* < 0.01.

**
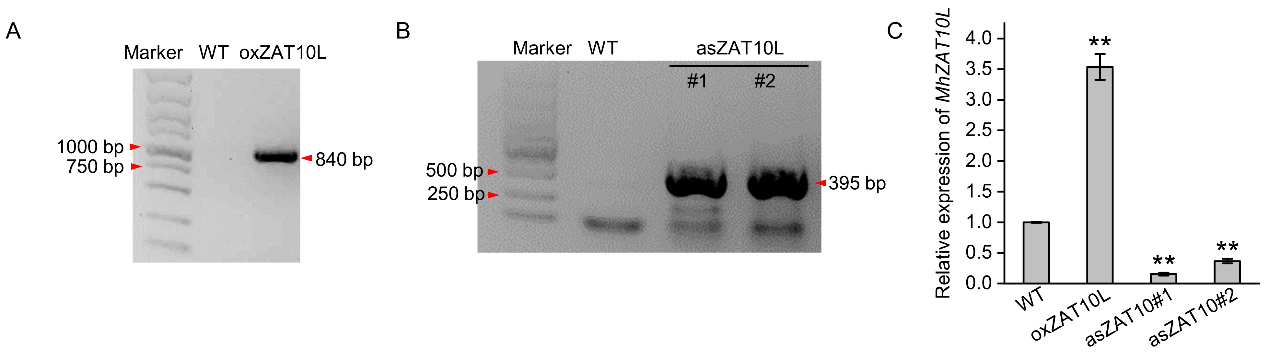
**

**Figure S6 Identification of *MhZAT10L* transgenic apple calli.**

**(A, B)** DNA strip analysis of *MhZAT10L* in *MhZAT10L* transgenic apple calli. **(C)** Relative expression of *MhZAT10L* in *MhZAT10L* transgenic apple calli*.* The primers used for DNA strip analysis were the 35S promoter primer and the gene reverse primer. The data are presented as means ± standard deviation (n = 3). All asterisks above the column denote significant differences detected by a two-tailed Student's *t*-test: ***P* < 0.01.

**
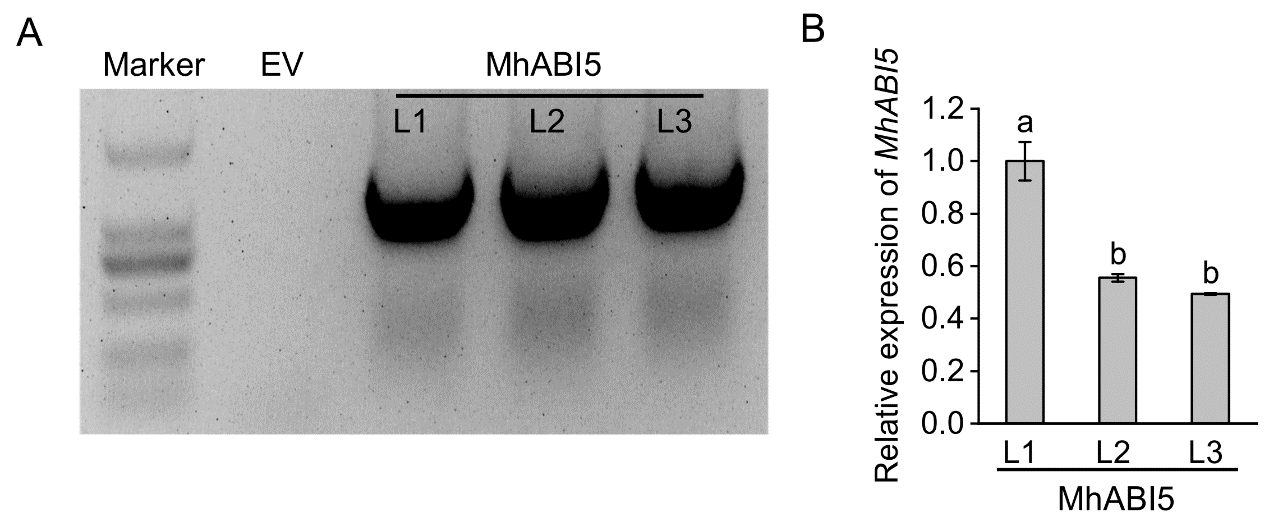
**

**Figure S7 Identification of *MhABI5* transgenic Arabidopsis.**

**(A)** DNA strip analysis and **(B)** the relative *MhABI5* expression in *MhABI5* transgenic Arabidopsis. The data are shown as the mean ± standard deviation (n = 3). Statistically significant differences (*P* < 0.05) are indicated by different letters in each column (one-way ANOVA).


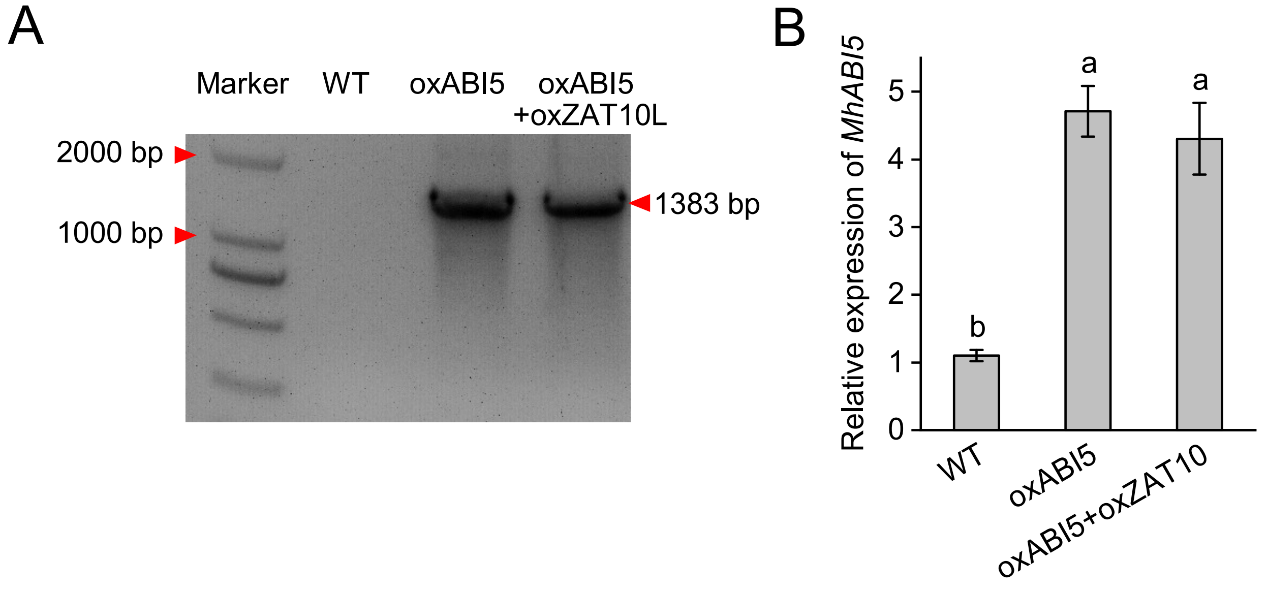


**Figure S8 Identification of *MhABI5* transgenic apple calli.**

**(A)** DNA strip analysis and **(B)** the relative *MhABI5* expression in *MhABI5* transgenic apple calli. The fusion vector *35S::MhABI5-GFP* was transformed in *MhZAT10L*-overexpressing apple calli to generate *MhABI5* and *MhZAT10L* co-overexpressing apple calli. The data are shown as the mean ± standard deviation (n = 3). Statistically significant differences (*P* < 0.05) are indicated by different letters in each column (one-way ANOVA).
