# Supplementary material for: Abscisic acid regulates Cl− efflux via the ABI5-ZAT10-SLAH3 module in chloride-stressed Malus hupehensis
Source: Hortic Res. 2024 Jul 24;11(9):uhae200. doi: 10.1093/hr/uhae200 (PMC11387005; doi:10.1093/hr/uhae200)
Supplement: Web_Material_uhae200 [file web_material_uhae200.zip › Supplenmental Tables-HR-R1-5.15 ï1⁄4^cleanï1⁄4%.docx]

**Supplementary Tables for**

**Abscisic acid regulates Cl^–^ efflux via the ABI5-ZAT10-SLAH3 module in chloride-stressed *Malus hupehensis***

Jianfei Song, Junhong Yan, Baozhen Sun, Bing Chen, Xiaoyue Zhu, Hongcai Wei, Zhilong Bao, Fangfang Ma, Weiwei Zhang*, Hongqiang Yang*

College of Horticulture Science and Engineering/Apple technology innovation center of Shandong Province, Shandong Agricultural University, Tai’an, 271018, Shandong, China

***Correspondence:**

Hongqiang Yang ([hqyang@sdau.edu.cn](mailto:hqyang@sdau.edu.cn)); Weiwei Zhang ([zhangww@sdau.edu.cn](mailto:Zhangww@sdau.edu.cn))

The PDF file includes:

Table S1 to Table S9

| **Gene ID** | **Gene name** | **annotation** |
| --- | --- | --- |
| LOC103448908 | *MdCYP72A219L* | *Malus* x *domestica* cytochrome P450 CYP72A219-like |
| LOC103446093 | *MdFOX2L* | *Malus* x *domestica* flavin-dependent oxidoreductase FOX2-like |
| LOC103455904 | *MdZAT10L* | *Malus* x *domestica* zinc finger protein ZAT10-like |
| LOC103404758 | *MdENT3L* | *Malus* x *domestica* equilibrative nucleotide transporter 3-like |
| LOC103428743 | *MdSOD [Cu-Zn]* | *Malus* x *domestica* superoxide dismutase [Cu-Zn] |
| LOC103442984 | *MdCAB151* | *Malus* x *domestica* chlorophyll a-b binding protein 151 |
| LOC103409034 | *MdPGK* | *Malus* x *domestica* phosphoglycerate kinase |
| LOC103405819 | *MdTHO4DL* | *Malus* x *domestica* THO complex subunit 4D-like |
| LOC103423136 | *MdCP12-1* | *Malus* x *domestica* calvin cycle protein CP12-1 |
| LOC103428089 | *MdPsbR* | *Malus* x *domestica* photosystem II 10 kDa polypeptide |
| LOC103444255 | *MdDJ1DL* | *Malus* x *domestica* protein DJ-1 homolog D-like |
| LOC103445243 | *MdLAO* | *Malus* x *domestica* L-ascorbate oxidase homolog |
| LOC103449726 | *MdUK2* | *Malus* x *domestica* uanylate kinase 2 |
| LOC103435885 | *MdceQORH* | *Malus* x *domestica* chloroplast envelope quinone oxidoreductase homolog |
| LOC103405066 | *MdRRP15L* | *Malus* x *domestica* RRP15-like protein |

**Table S1 Gene information obtained from a Y1H screen using the *MhZAT10L* promoter.**

**Table S2** **Primers used for vector construct and gene expression analyses in the study.**

| **Primer name** | **Primer sequence (5′- 3′)** | **Function** |
| --- | --- | --- |
| *MhSLAH3*-F1 | ggacaagggtctagacccgggATGATGGATAAAAGCAAAATTTTGG | Inserted into pBI121 |
| *MhSLAH3*-R1 | tctatcgatcaatcaggatccTAGCTTGGCATCTTCTAAATCTTTATTT |  |
| *MhZAT10L*-F1 | ggacaagggtctagacccgggATGGCTCTGCAAGCTCTCAAC |  |
| *MhZAT10L*-R1 | tctatcgatcaatcaggatccATTTTGTTGGTCCTGTGAAGTCTC |  |
| *MhABI5*-F1 | ggacaagggtctagacccgggATGTGCTTCAATTTGATCGGG |  |
| *MhABI5*-R1 | tctatcgatcaatcaggatccCAAACCACAACTATGACTCCTC |  |
| *MhSLAH3*-F2 | ggggacaagtttgtacaaaaaagcaggcttcATGATGGATAAAAGCAAAATTTTGG | Inserted into pGWB405 |
| *MhSLAH3*-R2 | ggggaccactttgtacaagaaagctgggttTAGCTTGGCATCTTCTAAATCTTTATTT |  |
| *asMhSLAH3*-F | ggggacaagtttgtacaaaaaagcaggcttcTGACTTTTGGCAGTTCGCTG |  |
| *asMhSLAH3*-R | ggggaccactttgtacaagaaagctgggttAGCCAAGTGACCTTCAACCAA |  |
| *MhZAT10L*-F2 | ggggacaagtttgtacaaaaaagcaggcttcATGGCTCTGCAAGCTCTCAAC |  |
| *MhZAT10L*-R2 | ggggaccactttgtacaagaaagctgggttATTTTGTTGGTCCTGTGAAGTCTC |  |
| *asMhZAT10L*-F | ggggaccactttgtacaagaaagctgggttCTGTTTGCGACAAGGGCTTC |  |
| *asMhZAT10L*-R | ggggacaagtttgtacaaaaaagcaggcttcGAAGAAGTCGCGGGAGAACT |  |
| *MhABI5*-F2 | ggggaccactttgtacaagaaagctgggttATGTGCTTCAATTTGATCGGG |  |
| *MhABI5*-R2 | ggggacaagtttgtacaaaaaagcaggcttcCAAACCACAACTATGACTCCTC |  |
| *asMhABI5*-F | ggggaccactttgtacaagaaagctgggttGAATGGGGCTTACCCGACAG |  |
| *asMhABI5*-R | ggggacaagtttgtacaaaaaagcaggcttcGAATGGGGCTTACCCGACAG |  |
| *MhZAT10L*-F3 | tatcggaattaattcggatccAATGGCTCTGCAAGCTCTCAA | Inserted into pET22b |
| *MhZAT10L*-R3 | gtggtggtggtggtgctcgagATTTTGTTGGTCCTGTGAAGTCTCG |  |
| *MhABI5*-F3 | tatcggaattaattcggatccAATGTGCTTCAATTTGATCGGGT |  |
| *MhABI5*-R3 | gtggtggtggtggtgctcgagAAACCACAACTATGACTCCTCC |  |
| *MhZAT10L*-F4 | tcccccgggctgcaggaattcATGGCTCTGCAAGCTCTCAACT | Inserted into pGreen II-62 SK |
| *MhZAT10L*-R4 | gggccccccctcgaggtcgacATTTTGTTGGTCCTGTGAAGTCTCG |  |
| *MhABI5*-F4 | tcccccgggctgcaggaattcATGTGCTTCAATTTGATCGGGT |  |
| *MhABI5*-R4 | gggccccccctcgaggtcgacAAACCACAACTATGACTCCTCC |  |
| *MhSLAH3pro*-F | agcttACATTTATATGTAAAATATAATTTGTTCACTTAACTAAGCATGTATTAg | Inserted into pGreen 0800-LUC |
| *MhSLAH3pro*-R | gatccTAATACATGCTTAGTTAAGTGAACAAATTATATTTTACATATAAATGTa |  |
| *MhZAT10Lpro*-F | agcttTCTCCGAAATTATCTAGTAATACGTGTGTGAAGGGATCGAAAGCACCAGg |  |
| *MhZAT10Lpro*-R | gatccCTGGTGCTTTCGATCCCTTCACACACGTATTACTAGATAATTTCGGAGAa |  |
| *35S* promoter | GACGCACAATCCCACTATCC |  |
| *qMhSLAC1*-F  *qMhSLAC1*-R  *qMhSLAH1*-F  *qMhSLAH1*-R  *qMhSLAH2*-F  *qMhSLAH2*-R  *qMh/MdSLAH3*-F  *qMh/MdSLAH3*-R  *qMhZAT10L*-F  *qMhZAT10L*-R  *qMhABI5*-F  *qMhABI5*-R  *qMhVPEγ*-F  *qMhVPEγ*-R  *Mh/Mdactin*-F | ACGGACAGTGGCTTTCGG  GCCTCATCCCACCCAACC  GTTCGGAGGCCAGCGTAG  ACGCCCCCACAAAGTTCC  GCCACCGTCACCAATGGA  TGCTGCCGCTGATCTCAG  CAGCCAAGTGACCTTCAACCA  AACGCTTTGATGAATTCGGGG  GGCTCTGCAAGCTCTCAACT  TTCTTTAGTGGCTGTGGCGG  GAATGGGGCTTACCCGACAG  CCCAGAGTTCTCAACCGGAC  CAGAAGAGAGCAGTTGGGGG  TCACTGTCTTCCATCCACGC  TAAGGCTGGATTTGCTGGAG | qRT-PCR |
| *Mh/Mdactin*-R | GCATCTTTCTGACCCATTCC |  |

**Table S3** **The *MhSLAH3* sequence without the termination codon.**

ATGATGGATAAAAGCAAAATTTTGGATACTGAGAAACAGAATTCTATTGAACTTCCAGCTCTCATCAAGCATATATCATCAAATGAAGTGGCTGGCTTTGATAATGTTGAGGAGACTATATTTCTACAGCCAAGTGACCCTCAACCAATCAGTCCATCAGCTAAAGGAATTGAAACGGCTGATTTAGAAAGCGTAGTTGATGAATCTGATCCCCGAATTCATCAAAGCGTTTATTCTGTTTCTATTAGCATGCCACCATCACCAATGGAAATTCATTTACAGAATAGCAAAAAGATGATGTTCAGTGGCGAAACAAGTTTCAACAATGGAATTCCAAATTCTTCCGCTGAGACTGAGAGTGCTGGCAGCGAACTGCCAAAAGTCATAAAATGTCACTCTCAGCCAATACTTCATAGCTCTGCCCTTGAGGAGGCAGTTAGCACTGGATGTATTTCTTATCATCCAGGTGTTGAAAGGTTGATAGATAGAAGGTTTGATACTTTCAAAACATGGTCCTGGAAGCTTGATAGGCAGACACCAAGTGCGGCGCAAGCAAAGAATGGTAATAATGCACAAAATGTAGAAATTGAACGTTTGCCTGCAGACCGATACTTTGATGCACTCGAAGGGCCAGAGTTAGACACCCTTAGGGATTCAGAAGAAATACTCCTTCCAGAAGACAAACAATGGCCATTTCTTCTCCGGTATCCAGTTTCTTCATTTAGCATATGCCTCGGTGTTAGCAGCCAAGCAATTTTGTGGAAGGTCCTTCCTACCTCATCCTCCACAAAATTTCTCCACTTAAGCTTAACACCAAATCTAGTTCTGTGGTGCATTTCTGTTGCTCTTGTAGCTATGGTTGCTTGTATATACCTTCTAAAAGTGATCTTTTACTTTGAAGCAGTTCGTCGTGAGTATTTCCACCCAATTCGTGTCAACTTCTTCTTTTCCCCATGGATAGCCCTCTTGTTCTTAACTCTTGGAATACCACCTTCATTTGCAAACAAACTGAACCCAGCTATTTGGTACATCCTTATGACCCCAATCTTATGCCTCGAGCTTAAAATCTATGGACAATGGATGTCAGGAGGCCAACGTAGGCTTTCAAAGGTAGCCAATCCTGTTAACCATCTAGCAATTGTCGGGAACTTTGTGGGAGCATTGCTAGGCGCATCAATGGGACTAAAAGAAGGACCAATTTTTTTCTTTGCTGTTGGGTTGGCTCACTACGCAGTTTTATTTGTAACTCTCTACCAGAGGCTTCCAACAAATGAAACTGTAATCCCAAAGGATCTTCATCCTGTATTCTTTTTGTTTGTTGCAGCACCAAGTGTAGCTTCCATGGCATGGGCAAAAATCCATGGCTCCTTTGGTTACGGTTCACGAATTTTATACTTCATTGCTTTGTTCCTTTATCTCTCACTGGCAGTTCGGGTTAATTTCTTCAGAGGATTTAAGTTTTCTTTGGCATGGTGGGCATATACTTTCCCGATGACTGGTGCTGCCATTGCAACCATCAGGTACTCGAACGAAGTCACGAATCCGGTAACACAAGCTCTGGCTGTCATACTCTCTCTCACTGCCACGATCATAGTCACTATTCTCCTCATAGTAACTATATTGCATGCCTTTGTGCTCCAAGACCTCTTCCCCAATGACATTGCAATTGCCATCAGTGATAGAAGTCCTAAACTAAACAAGAAGTGGTTCCACCTCAGACATGGAAGCTCGGATACCAGGGACATTGACAAATTCTTGAAATCAACAAGAAGTTTAGAAAATAAAGATTTAGAAGATGCCAAGCTA

**Table S4** **The promoter region of *MhSLAH3* (2000 bp from the upstream ATG).**

GAAGAGAATGCACTTGCTGACAAATTAGTTAATTTAGGACTTCTTTCGTCTTCACTTGTTTGGCAATGCGCTTGTCCTACAAAGATTCTTCCTTTTCTACATTCAGATTTCTTGGGCATGCTAGCCTACAGGTTTATCTCTCCTTCTTGGTGTGTGATTTTCTCTTTTACCTAACCCTTTTGGATTTTCTTTTTGTTTTGCAGCATGTTTTTATCTTTAGGTTTTGTTTTGGTCCTATTCCCTCCCTTTTATTGGTATTTTCTTTTTCAAGAAGGGTAAGATTTGTCCCCTCTCTTTTCTTGGTATTTTCTTTTTCAAGAAGGGTAAGGTTTTTTCCTATATTTTGGTTTTCATATCAATAAAATTCCCTCTTATTGTGAAGATTTAGAAAAAAAAACGTGTAGGTTGGCATCGGTGTAGTTTTTAGGGTTATTTAGATATAGACTCTTGTAACTCTTATTTCCTATATAAAAACCCACCCAATTATACACATTCAAATAAAACCCAAATTTAAAAGTGATTGCCATGTAAGAAAGTAATTGACCTATTAATACAAAATATTTACAACTTGTACCACAACATTCAAAATAAATCAATAAATATTATTACTCACCTTAATTACAATGAACAAATAAGTTCTTGCATATTATTACCCCTAACACACACACACACACACACATATATATATACACATATATACGTTCAAATGTATATAGTACTACCACAAGCTCAATATATATATGTATGTACGTATGTATGCACATTACTACATATATGATCAAAGATATTATACTAATTAATTTAATCTACCTATATGTAAATTTATGATGAGCAAATAACGTATATTTTGTAGGTATATTATTTTGTATGTATCATCATATATATTGGGCACATTTTATTATTATATGAACAAAATAATAGGTAAACTAGTATTGAATAAAATAATAATATTTTTTTATGTAGGTACATTATTTTACATACATTGGGTTTGCTTCATAATGTAGGTAAATTATTTTNCATGTATTTTACATACAGTGGGGAAATTATTTTGCTTTTTTTAAGCAATCTAACATTTTAAAATTCGAATAGTAGGTGCACTGAATCAAATTACATTTTTTGTAGGTACATTATTTTGCATACATTGGGTTTGCTTTATTATGTAGGTAAATTATTTTGCATGTATTTTACATACAGTGGGGAAATTATTTTNCTTTTTTTAAGNAATCTAACATTTTAAAATTCGAATAGTGGGTGCACTAAATCAAATAACATTTTTTGTAGGTAAATTATTTTGCACAAATTTTTACGTATACCAGACATTTTTTTTATTTTTTATTTTTGTTATATATATGTGTGTGAAATAATTTACCTACATTTATATGTAAAATATAATTTGTTCACTTAACTAAGCATGTATTATTACATATATTAGGCATGTTTTGTTGTTATTATATATTAGGCAATAAATTTATTATTTATTATTTTTCGTAAAATCATTAATTCTCACTTACAATCTATATGGAACTAATTGTGTACATCAAACATTCAAATAGGGGTGTTTTGGGCGTTAAATTTTTTTAAATGTGTAAAGTCAAATATAATTAATGAATTTGCTGATGTGGAATCTAGTTAATGGGTTTTATTTGAGTAAAAAACTTATGACGGGTTTTATGTGGAGAAAATTAAATTTCAGGGGCTAAAGTCATATTTTCAGAAGTTTTTATTAGCTTGGTTTGACATATGTGTATTTACTTGTAATGCAATTAATAAAATGAATTTTTTACTCATGAGTCGTGAAACATTTTTCATCACATTATTTATTTGTTCAACGATTAGTATAAAAAACTTGATATCACATTCAAAGCACACCATGTTTGAAGTGCCTATATATACATGATAAATTTTTAATTACACTGCACCAGAAATGAGTGGTCATCTGTTTATGTACTGCAGATTGAGCTGCATTTCTTAACGTTTTCTGGTTTAATTTTTTTTAAATG

**Note:** The highlighted base sequence is the binding site for MhZAT10L.

**Table S5 The *MhZAT10L* sequence without the termination codon (accession number, MZ540769).**

ATGGCTCTGCAAGCTCTCAACTCGCCAACAGCCGCCGGACCCTCTCCTTTCCCATTTGAGAACGAGGCCTCCAGCCTCGGCTATGCCGAGCCGTGGACCAAACGCAAGCGTTCCAAGCGTCCACGCCTCGACACCCCTCACTCCGAGGAGGAGTATCTTGCCATCTGCCTCATCATGCTTGCCCGCGGAAACAGAGGTGGAGACCTCGCCTCTACTACCGCCACCACCGCCACAGCCACTAAAGAAACAAACTCCGCCGCCTCACACCAGATCATCACTCAATCCCCTTCTATGGAGCCCTCCACGTCAGCATCTCCGCCGGTTAAGCTGAGCTACAAGTGCTCTGTTTGCGACAAGGGCTTCTCTTCCTACCAGGCGCTCGGAGGACACAAGGCCAGCCACCGGAAAGGCTCTGCAGCCGGATCGGCCATCGAAGGCCCGTCCACGTCATCAACCACAACAACCTCTGCCGCGGCCACCGCCACCGCTTCCGGTAGGTCCCACGAATGCTCCATCTGCCACAAGTCTTTCCCCACCGGTCAAGCCTTGGGCGGCCACAAGCGCTGCCACTACGATGGCGGCGCCGCCGGATCCACAGCCACCACCACCGCTACCACGAGCGCTATAACTTCCTCTGAGGGAGTTGGGTCAACTTCTCACGCGGTCAGCCACGGCCACCCCCGAGAGACGTTTGACCTGAACCTGCCGGCGTTGCCCGAGTTCTCCCGCGACTTCTTCATCTCCGGCGAGGACGAAGTGGAGAGCCCCCACCCGACGAAGAAGCCCAGGCTTCTACTGATGATGAAGCCCAAAACCGAGACTTCACAGGACCAACAAAATTAG

**Table S6** **The promoter region of *MhZAT10L* (2000 bp from the upstream ATG).**

AATGAGTCTTGATCATACAACTACGTGATACAAGCGTCCAAATCAAGAAACCTCTCGTAAAGTGAACCAAAATAATGCGTTAGTAGTACTACATTTTAAGCAAATTTAGATAGCATTTATCCTTTCATTTAGCAGTTAGCAAAATTTCATTTGTGTGATTGTGGAATGCTCATTAGGTCATTGAGAAAATTAATGCTAACATGATGAGATACCAATCAAAGTTTTTATGGCATTTGTGATAGGAAGGCTAATTAAAGTAACGTGATATAAGATTTTCTATAGTAATCAGATTCCAAAGCCCAATTAAAGACAAAGATTTCCCCAACCCTTGCAAATGGCGTGCCTAAAATTAGGCTTTGCACAAAAGACCCCACGCCCACAGTCTCTGTGCACCACTAGTTAAGCACAGTTAATCTCCACCACACTAACGAGACATCACATGTGATTAGGCCCTTAGTCACCCCCTAACTAAGCAATGACGGTGGTGTTTTGATTGAGATTTTAAGATGCCAACTTCCAACCGGATAAACTCTCCGAAATTATCTAGTAATACGTGTGTGAAGGGATCGAAAGCACCAGGAAAAAGTCGCTGTGCTTCCTTGAATGTGGGGGATCAATACGACGTCGGCTTCTTCCGATTGGAAATTGGAAATTAGATTGGACTAAAAGCCTTTTATTTATTTTTTTAAATTTTTTTTAAGTACATCGATATATTTACACTGGGAGAAAGGAGAGTTCGACTAAGCCGCACAATGTGCAATCTAATTTGATATCGAATTCACCATTCATGATATTCGAATCTAAGATCTCTCACTTTCGAATGAAAAGAGATACCATCAGACCGTAGTACTGACCGCATTTATTATCTTTTCCAATCTTATAACAGATTTTAAAATAAGGAAAACTAATGAAAAATGCTTGAAAACTTTGAGTTTTAATGATAAGGATAAAATAAAGGGTAAAGTGAATAGTACCAAGATTGATTTTTTAGTGTAAAAATGTGGTTTTTCGTTAAAGTGAACAGTACCGGATGCTTTTCATTAAAGTTTCCTTTAAAATATCCAAACCTATATGATGGAAATTAAATTACGTATAAAATTACATGCATAGTATTATGTTCTGCTGATTTAAATTATATTTTTTTATCTGTAAATTTTACAAATTAAATTATGTACGCTATAATCCAAGCAACTTAATGTGCAAAATTAAGACGAACAAAACCCGCAACAACATACTTAATCCCAAAATATTATAAGAAAACTAACGAAAAAAGTTTGACAACTTTGAGTTTTAACGATAAAGACAAAATAAACGGTAAATTGAATAGTATCATGATTAACTTTTTAGTATAAAAATATAATTTTTTCATTAAAATAAACAGTACTACAAGTTTTTTTGTTAAAGTTTCCAATATTAAATGTAATCGTACTAACACATGATAATCAGTATCCGGACCGCGTTTATTTAGAAATTAGAAGATTTAGAAAGTGTCCACTCTCTCCAGACCCTTGACTTTGAAACCGTCTTTGGTGTTTGAGCCCCCAACAGAAACCCTAAAATTACACGTAAAAAGTCTTCTGTAGTTAAAAAGTTACCCAAAAAAAAACAAACAAACCGAGTCCAGTGTGGGCCCACGCACCCAGAGGGGCACCACTTCAACTGTTTGACCGACTTCAGTCCTCCTTGGCCACCAAGAAAACTCAACTCAACCTCCCCCCACCCAAATCTGAACGTGGTAAAGTTCCTCAAGTCTCTCTCTAATTTTCCGAAATAATAAAAAATAGAACATATGATATTTATTTAAATACGGACACTTACTTCTACCACTTGTCACGCAACTTCCCCTCTCTTCCTCGTCCCTTTATATATACCCCCCATTCCCCTACTTCCACTCTCACTTCCAACTCTCAACTCTCAATTCTCTCAGCTCTCTCTCCTCCCATTCTCTCTCTAAAAACAGAAAACAGAACCTCTTAAAATTTTTTATCAACCATCATCAG

**Note**: The highlighted base sequence is the binding site for MhABI5.

**Table S7** **The *MhABI5* sequence without the termination codon.**

ATGTGCTTCAATTTGATCGGGTTCATTACAATTTTGAAACCCATCATTCAATCAGGTGTAGATCACACAAACATGGGTGTTTCGGAGTCGGAAATCATCTCCCAAGGCAAGGTGGATTCGCCTTTGTTATCGGACCAACAAGAGAAGAACCAGTTATTCTCTTCATTAGGAAGACAATCATCAATCTACTCTCTCACTCTTGATGAGTTTCAGCACACCCTCTGCGAGAGTGGCAGGAGTTTTGGGTCGATGAATATGGATGAGTTCCTCACAAGCATTTGGACCGCCGAAGAAAACCAAGCCATTAATTCCAATCACACCAACACTTCCACTACTAACAACAACACGAACAACATTGAGGTGCACATGCCTTTAGCCGATGCCTCTGCAGAGAAGCACATTGCGACGCAGCCCAGCTTGCCGCGACAAGGCTCACTCACACTGCCTGCGCCACTGTGTAGGAAAACGGTGGATGAAGTTTGGTCCGTGATACACAAAGGGCAGCAGGCAAAGCAGCAGAACAATCACAACGGCAGCATCGACGGCGGTGCTCAGAGTTCTGAGTTTGCCCCTCGTCAGCCTACTTTTGGGGAGATGACATTGGAGGATTTTTTGGTTAAAGCAGGGGTAGTTCGGGAACAGGATTCAATGGCAGCCACAGTGGTGCCTCCTCAGCCTCAGCAGCAGCAGCAATATGGGATGTATCAGGACGGCAACCAGGCAGTGGTACCCAGTTTTGTTAATAGGCCTGTGATGGGAATGGGGGCTGCTGGTGCAGCAGGTACTAGCACTGCTACCGGTATTCCTAATTACCAAACTATACCACAAAGTGGGTCTGCGGTTGTGGGAGAGTCCTCTGCGTATGCTGCGAATGGTAAGAGGAATGGGGCTTACCCGACAGTGCCACCTCCACAGTCGGTTTGTTTTGGCGGGAAAGTGGTGAATGGTGGTGATGGATATGCAGCAGGGCAGACAATTGGGATGGGGGCTCTTGTGAGTCCGGTGTCTTCTGATGGGATGGGTACTAGTCCGGTTGAGAACTCTGGGGGTCAATTTGGTTTGGAAATGGGTGGACTAAGAGGAAGGAAGCGGGGTTTAGATGGAGCGGTCGAAAAAGTGGTAGAGAGGAGGCAGAGAAGGATGATTAAGAACAGAGAGTCTGCAGCAAGGTCTAGAGCCCGAAAACAGGCATACACAGTTGAATTGGAAGCAGAACTGAACCAATTGCGAGAAGAGAACTCACACCTTAAACAGGCGCTGGCAGAGCTCGAGAGGAAACGAAAGCAACAGTATTTCGAGGAAATGAAGATGAGAGTTCAGAAGAGGGCCCAGAAGGTGAAGGAGAAGCTAAGGGTGTTGAGGAGGAGTCATAGTTGTGGTTTG

**Table S8** **Putative ABI5 binding sites.**

| **Matrix ID** | **Sequence logo** | **Species** |
| --- | --- | --- |
| TFmatrixID_0187 | 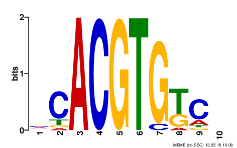 | *Arabidopsis thaliana* |
| TFmatrixID_0846 | 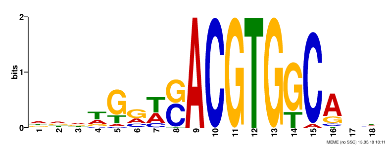 |  |
| TFmatrixID_0849 | 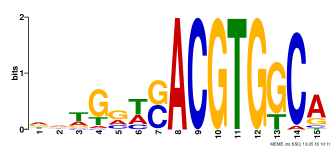 |  |
| TFmatrixID_0182 | 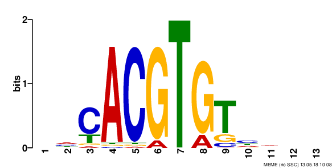 | *Glycine max* |
| TFmatrixID_0183 | 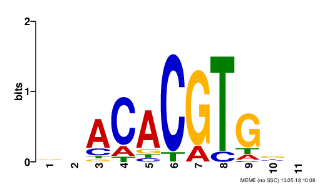 |  |
| TFmatrixID_0184 | 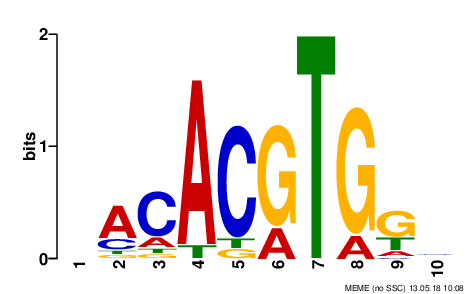 |  |
| TFmatrixID_0193 | 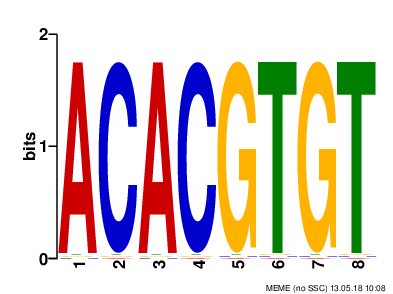 |  |
| TFmatrixID_0515 | 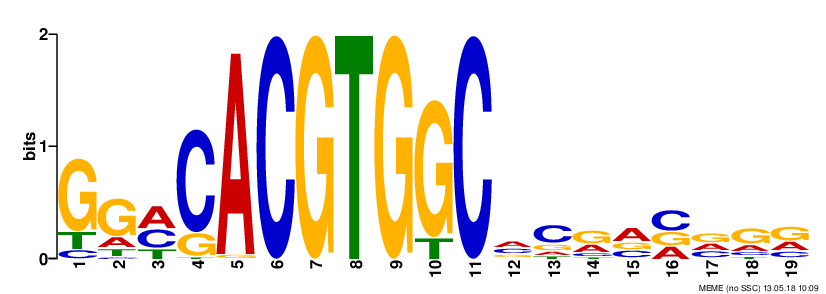 |  |

| **Matrix_ID** | **Position** | **Strand** | **Similar Score** | **Hit sequence** |
| --- | --- | --- | --- | --- |
| TFmatrixID_0182 | 549 | + | 0.96 | aatACGTGtgtga |
|  | 1464 | - | 0.96 | aattaCACGTaaa |
| TFmatrixID_0184 | 549 | + | 0.93 | aatACGTGtg |
|  | 1728 | + | 0.92 | tgaACGTGgt |
| TFmatrixID_0193 | 30 | + | 0.75 | ACAAGcgt |
|  | 436 | - | 0.75 | tcaCATGT |
|  | 438 | + | 0.75 | ACATGtga |
|  | 550 | + | 0.88 | ATACGtgt |
|  | 550 | - | 0.88 | ataCGTGT |
|  | 552 | - | 0.75 | acgTGTGT |
|  | 1386 | + | 0.75 | ACAAGttt |
|  | 1432 | + | 0.75 | ACACAtga |
|  | 1468 | + | 0.75 | ACACGtaa |
|  | 1824 | - | 0.75 | ccaCTTGT |

**Table S9** **Potential binding sites of ABI5 on the *MhZAT10L* promoter.**
